# Associative Vocabulary Learning: Development and Testing of Two Paradigms for the (Re-) Acquisition of Action- and Object-Related Words

**DOI:** 10.1371/journal.pone.0037033

**Published:** 2012-06-06

**Authors:** Nils Freundlieb, Volker Ridder, Christian Dobel, Stefanie Enriquez-Geppert, Annette Baumgaertner, Pienie Zwitserlood, Christian Gerloff, Friedhelm C. Hummel, Gianpiero Liuzzi

**Affiliations:** 1 Department of Neurology, University Hospital Hamburg-Eppendorf, Hamburg, Germany; 2 Psychological Institute II, University of Muenster, Muenster, Germany; 3 University of Applied Sciences Fresenius, Hamburg, Germany; 4 Department of Neurosurgery, University Hospital Zurich, Zurich, Switzerland; Utrecht University, Netherlands

## Abstract

Despite a growing number of studies, the neurophysiology of adult vocabulary acquisition is still poorly understood. One reason is that paradigms that can easily be combined with neuroscientfic methods are rare. Here, we tested the efficiency of two paradigms for vocabulary (re-) acquisition, and compared the learning of novel words for actions and objects. Cortical networks involved in adult native-language word processing are widespread, with differences postulated between words for objects and actions. Words and what they stand for are supposed to be grounded in perceptual and sensorimotor brain circuits depending on their meaning. If there are specific brain representations for different word categories, we hypothesized behavioural differences in the learning of action-related and object-related words. Paradigm A, with the learning of novel words for body-related actions spread out over a number of days, revealed fast learning of these new action words, and stable retention up to 4 weeks after training. The single-session Paradigm B employed objects and actions. Performance during acquisition did not differ between action-related and object-related words (time*word category: p = 0.01), but the translation rate was clearly better for object-related (79%) than for action-related words (53%, p = 0.002). Both paradigms yielded robust associative learning of novel action-related words, as previously demonstrated for object-related words. Translation success differed for action- and object-related words, which may indicate different neural mechanisms. The paradigms tested here are well suited to investigate such differences with neuroscientific means. Given the stable retention and minimal requirements for conscious effort, these learning paradigms are promising for vocabulary re-learning in brain-lesioned people. In combination with neuroimaging, neuro-stimulation or pharmacological intervention, they may well advance the understanding of language learning to optimize therapeutic strategies.

## Introduction

Recent neurophysiological research has extensively advanced our knowledge of linguistic operations in the brain. These include widespread brain systems engaged in understanding word meanings, but to date, little is known about the neural mechanisms involved in the acquisition of a novel vocabulary, or the recovery of language after brain damage. Given the high incidence of post-stroke aphasia [1], [2], language (re-)acquisition is a central topic in current neuroscience. Moreover, re-acquisition may be different for different classes of words, depending on what they refer to [3]. It has been proposed that neuroscientific evidence may translate into efficient neuroscience-based rehabilitative training. However, robust language learning paradigms compatible with up-to-date neurophysiological techniques are scarce. Therefore, the aim of our study was to adapt and test two learning paradigms, using novel words for actions and objects.

The developed paradigms rely on two principles that may well be important for effective aphasia therapies: massed practice and associative learning [4]. According to the Hebbian theory, associative learning requires concurrent firing of two neurons or neuronal populations in order to strengthen the neural connections between them. The newly wired connections then allow more efficient transmission from one neuronal population to another, which is regarded as the neural correlate of learning ([5]; “fire together, wire together”). The paradigms used here are based on Hebbian assumptions of associative learning, and rely on massed practice. They aim at establishing functional links between lexical and semantic information by frequently co-activating brain regions processing lexical and semantic information [6]. If a brain lesion has weakened the connection between a word and its meaning, language therapy based on repeated associative learning may strengthen remaining links or establish new ones, and thus improve performance [7]–[9]. As mentioned before, (re-)acquisition of words may be different depending on word class – and thus on types of concept. Recent models postulate tight links between perception-action systems and the language system to establish word meaning [8], [10], [11]. The meaning of action-related words is supported by activation of the relevant motor system. For example, processing words such as “kick” activates leg motor areas, whereas “lick” activates brain areas controlling tongue movements [12]–[14].

Previous studies also demonstrated that novel object-related words can be acquired with associative learning [15], [16]. Combined with functional imaging techniques and non-invasive brain stimulation, such paradigms have substantially contributed to the understanding of neural correlates for the learning of novel words for objects [17]–[20]. However, the mechanisms and the brain regions involved in learning action words may differ compared with object words [21].

To establish robust learning paradigms for studying action- and object-related word acquisition, we developed and tested two different paradigms in young healthy adults. In a first step, pictures of actions and objects were rated regarding naming agreement, quality of depiction and recognisability. Pictures were then combined with pseudowords to form “correct” and “incorrect” picture and pseudoword pairings. These pairings were then repeatedly presented visually and auditorily to form training sessions. Then, participants had to intuitively decide whether pseudowords and pictures matched or not without receiving any feedback.

Paradigm A was based on a large set of action-related words. Learning was spread out over four consecutive days and retention was tested in three sessions up to 1 month after learning (day7, day14, day28). With Paradigm B, we directly compared the learning of object- and action-related words. Paradigm B tested the efficiency of learning in a single session divided into 5 blocks to suit neurophysiological research with time constraints [18].

In both paradigms, at the end of the training, participants were asked to translate the pseudowords into their native language. To exclude as many confounding factors as possible, participants were screened with an established neuropsychological test battery before being included in the study.

As previously demonstrated, associative learning of novel words for actions is achievable, and results in stable retention after a training-free interval, comparable to object-related words [15], [21]. The hypothesis related to Paradigm A was that learning of novel words for transitive and intransitive actions differs even when word properties are balanced. The hypothesis related to Paradigm B was that learning of action- and object-related words differs even when learning conditions and quality of stimuli are equivalent.

As outcome parameters, we measured the rate of correct translations, reaction times and correct decisions during training sessions and blocks.

## Results

### Rating

For Paradigm A, 76 actions were chosen based on high values for naming agreement, recognisability and quality of photographic illustration. Mean lexical frequency of action names (verbs) was 12.91±2.77 (which means 2^12^.^91^ times rarer than the most frequent German word “der” (“the”) as listed by the University of Leipzig “Wortschatz” (word frequency count, http://wortschatz.uni-leipzig.de). Each action was represented by four different pictures with different actors carrying out the same action, resulting in a total of 304 photos.

For Paradigm B, 17 actions and 17 objects were selected based on the same criteria as in Paradigm A. Mean lexical frequency was 13.35±2.76 (actions) and 11.24±2.79 (objects) (p = 0.033). Each object and action was represented by two different pictures, resulting in a total of 68 pictures. Naming agreement was not significantly different between action and object photos (object pictures: 95%; action pictures 96%; p = 0.12).

The selected materials are part of a much larger set of rated pictures, which facilitates additional cross-over studies that require several lexica (see Tables S1, S2 and S3 for rating results).

### Learning and Transfer

#### Paradigm A

**Figure 1:**
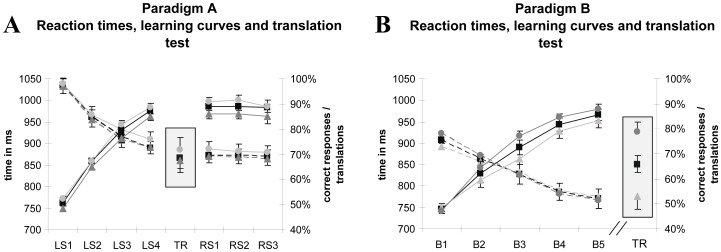
Results of Paradigm A & B. A Paradigm A Mean reaction times, learning curve and translation test. Subjects learn the pseudowords (correct and incorrect couplings) and retain them for several weeks. Please note that usually 50% is achieved by chance due to the design of the experiment. Translation test results are highlighted after the learning session on day 4; for single subject data see C. Reaction times within the experiment (starts 200 ms after pseudoword onset) shorten continuously as pseudowords are learned**.** Transitive actions are learned better. Dashed lines depict reaction times, continuous lines depict correct responses during learning. Please note that the transitive/intransitive results depict a subset of 21 items of each class. rectangles  =  overall (76 items) dots  =  transitive actions (21 items) triangles  =  intransitive actions (21 items) B Paradigm B Mean reaction times, learning curve and translation test Subjects learn the pseudowords in one session of 5 blocks. Objects are learned better than actions. Reaction times in Paradigm B drop like in Paradigm A analog to the learning process. Dashed lines depict reaction times, continuous lines depict correct response during learning. Translation test results are highlighted on the right, differences between word classes are more obvious. rectangles  =  overall dots  =  objects only triangles  =  action only Legend: LS: learning-session day RS: reassessment-session day B: Block TR: translation test.

Values of neuropsychological tests were within normal ranges for all subjects (for further details, see Table S4). The participants translated 68.8±4.3% correctly into German after the fourth training session (Fig 1A). Participants started at chance level of 50.7%±1.19% in session one (LS1) and reached a learning ratio of 87.5%±1.5% in session four (LS4) (Fig 1A).

rmANOVA showed a significant increase of correct responses over the course of training (time: F_6,108_ = 271.3; p<0.001), with significant differences between all learning sessions (LS1–LS4, all t-tests p<0.05), but no significant difference between the last learning session (LS4) and the first reassessment session (RS1), nor between reassessment sessions (RS1–3; all t-tests p>0.05). Subjects thus learned the new words during the learning sessions and retained this vocabulary at a stable level during the three weeks of reassessment.

Reaction times, measured from picture onset, also decreased significantly (time: F_6,108_ = 64.96; p<0.001) over learning sessions (LS1–LS4), and remained stable at reassessment (RS1–RS3).

#### Paradigm B

Values of neuropsychological tests were within normal ranges for all subjects (for further details, see Table S4). Subjects were able to translate 65.9%±3.4 of the words correctly after training. There was a significant difference in translation rate between action-related (mean = 52.9%±5.0) and object-related words (mean = 78.8%±4.04): t_9_ = 4.3; p<0.002 (Fig 1B).

The percentage of correct answers per block increased during training (time: F_4,36_ = 73.46; p<0.001; all t-tests between blocks p<0.05). Participants started at chance level of 47.7%±4.45% and reached 85.7%±7.38% in the last block. A two-way repeated measures ANOVA showed no significant interaction between time*word category: F_4,36_ = 2.49; p = 0.104.

Response latencies significantly decreased from block to block (time: F_4,36_ = 50.26; p<0.001); moreover, there was a significant interaction of response latency*word category (F_4,36_ = 4.71; p<0.05; ). Bonferroni-corrected post-hoc t-tests between reaction times for action- and object-related words per block showed no significant differences except for block 2 (Block 2: p<0.05; all other blocks p>0.05).

Given the significant difference in learning results for object- and action-related words, we asked whether a similar difference could be detected within the set of action words, when comparing transitive and intransitive words. We hypothesized different motor indices depending on transitivity, as recent research has shown differences in processing of transitive and intransitive actions [22]. We found significantly higher associations with head and whole body movements for intransitive compared with transitive verbs (head: t_40_ = 2.97; p = 0.005; whole body: t_40_ = 2.26; p = 0.03; arm, leg and motion association n.s.). Potential confounders (naming consistency, quality of depiction, distractors) did not show any differences regarding transitivity except for word frequency (t_40_ = 2.46, p = 0.02), with the intransitive words being more frequent than transitive words. Analysis of learning results revealed a significant main effect of “transitivity” (F_1,18_ = 39.21, p<0.001) and “time” (F_6,13_ = 61,84, p<0.001) and no interaction between the two factors (F_6;13_ = 1.07, p = 0.43). All sessions but LS2 showed a significantly different learning success (t-test for all but LS2 p<0.05). The translation-test showed no significant difference regarding transitivity (t_18_ = 1.37; p = 0.19).

## Discussion

The present study showed that novel words for actions and/or objects could be reliably acquired by associative learning without feedback. As expected by Hebbian rules, repeated co-occurrence of pseudowords and pictures of actions or objects led to a fast and stable acquisition of a new lexicon.

Based on extensive ratings, a total of 372 pictures of body-related actions and objects as well as 110 pseudowords were considered for the two paradigms, which were subsequently tested in 19 (Paradigm A) and 10 participants (Paradigm B). By varying the ratio of correct and incorrect couplings and the number of words to learn, similar learning success was obtained after four sessions on four consecutive days (Paradigm A) and after a single learning session (Paradigm B). The learning ratio was 87.5% in Paradigm A and 85.7% in Paradigm B. The level of correct responses remained stable over the course of 4 weeks, demonstrating a long-lasting retention for multiple-session training. Participants could explicitly translate the newly acquired vocabulary into their native language, with a mean score of 68.8% after the fourth learning session in Paradigm A, and a similar level (65.9%) after the single session of Paradigm B. Hence, repeated presentation of actions and objects with a new vocabulary conveyed the exact meanings of the novel words at the end of the training.

### Shaping of Language Learning for Particular Research Questions

In the presented paradigms, several parameters such as level of difficulty, time of acquisition, learning curve and retention over time can be precisely adjusted to fit a particular population or research question at hand. Therefore, these paradigms are well suited to investigate novel vocabulary learning in combination with neurophysiological techniques. In some studies, neuroimaging or non-invasive neurostimulation require a single session as in Paradigm B [17], [18]. Other language learning studies tested the effect of neuroactive drugs on long-term retention [23], for which a set-up like in Paradigm A is more suitable. Furthermore, by adjusting the ratio of correct-to-incorrect couplings as well as the absolute number of repetitions, even low cognitive resources as in brain-lesioned people can be accommodated. First and foremost, we demonstrated that learning of words associated with different types of concepts, such as actions and objects, was feasible within the same session, without provoking confounds. Hence, a learning setup as in Paradigm B is well suited to explore the neural mechanisms for encoding words associated with different meanings.

As mentioned earlier, associative word-learning paradigms already exist, but only for objects, often using one single line drawing per object [18]. To avoid the risk of learning mere picture-sound couplings, we used different photos with different actors and environments for the same action and objects. Each individual picture occurred once in a correct and once in an incorrect coupling. However, the coupling of the word with the correct action – implemented with different pictures - occurred more frequently than the incorrect couplings. By this distribution, the total number of occurrences for each picture, each word, each coupling and each type of coupling was equal. Only the ratio of concept to word (4∶2) allowed for learning the correct meaning of each word. Thus, subjects had to extract the concept (action or object) common to these pictures, and map the joint meaning onto the correct novel word. As shown by the results of the translation tests, subjects extracted the exact meaning of the novel words rather than only learning picture-word couplings. Finally, we monitored long-term learning for actions using four different photos for each action concept, and found similar learning results over time than earlier studies with single drawings for each object concept [e.g. 15].

To better suit patients with impaired cognitive function (e.g. stroke patients), we chose a paradigm with low attentional demands, based on frequent repetitions without explicit feedback. Beyond word learning, paradigms for structural aspects of language were developed based on artificial grammar learning (AGL, [24]). Although we used both words for actions and objects, we cannot draw any conclusions regarding learning of syntax. The presented paradigms were specifically designed to associate word forms with semantic knowledge of actions and objects. While AGL has been extensively explored [25], paradigms for vocabulary acquisition are sparse. To better understand the learning mechanisms of linking meaning to word form, the presented paradigms are suitable tools to be used for neuroscience-based research of vocabulary learning.

### Differences between Object and Action Word Learning

It has been proposed that processing pictures, extracting their common meaning, and relating this meaning to a novel word, is more demanding for actions than for objects. For example, semantic representations relating to objects are acquired before those relating to actions [26] It has been discussed that the semantic organisation is more complex for action concepts than for object concepts [27]. Also, actions have more complex perceptual features than concrete objects, and actions are more complex with respect to imageability than objects [28]. These factors may also explain why object-picture naming is faster and more accurate than action-picture naming in healthy and aphasic patients [27].

In Paradigm B, subjects were more successful in object- compared with action word learning. This effect can neither be explained by differences in the quality of the photographs nor by differences in recognisability. Rating for quality and naming agreement of actions and objects did not differ. Considering these rating results, alternative explanations are needed. In the light of the embodied cognition theory, we propose that different neuronal circuits are required to encode action- and object-related words. The hypothesis that learning of different word groups draws upon overlapping, but partly segregated brain networks is further substantiated by the behavioural results of our subgroup analysis for the action-related words. Transitive words were learned and retained significantly better than intransitive words, even when controlling for possible confounding factors. Together with the finding that transitive words had lower motor indices, this could be indicative of a weaker representation within the motor system. These findings give rise to the idea that more complex motor associations require more sophisticated neurobiological resources, e.g. more widespread connections with the motor cortex [29]. In keeping with these findings, interference with motor cortical involvement has been shown to influence acquisition of action-related words, but not object-related words indexing different neural substrates for learning different word categories [21].

It seems that acquisition of words has substantially different neurophysiological groundings, even within the same category of words, e.g. transitive and intransitive verbs. However, interpretation of the subclass analyses has to be taken cautiously at this point, since the study was not a priori designed for the investigation of word subclasses and the specific impact of motor association on learning. Hence, further studies are needed to test this novel hypothesis. Other types of verbs might be acquired in a different way. Mental-content words such as “think” or “believe” are particularly difficult to learn. It has been suggested that specific contextual information such as “false beliefs” together with linguistic cues are important to build a vocabulary of mental verbs (Papafragou et al., 2007).

### Options and Limitations

First, it should be explicitly stated that we did not intend to develop a therapeutic or diagnostic approach to aphasic rehabilitation. Nonetheless, in a proof-of-principle-study, Breitenstein and colleagues [30] showed successful learning with an implicit language-learning paradigm in aphasic patients. Thus, such paradigms – including the ones presented here – can be adapted for monitoring and testing vocabulary re-learning in aphasic patients.

Both paradigms are well suited to (a) study underlying mechanisms of lexical acquisition with modern neuroimaging and (b) to evaluate effects of novel innovative interventional strategies, such as non-invasive brain stimulation [31] or pharmacological interventions [32], [33], to enhance re-acquisition of language skills after impairment due to brain pathology. The paradigms are easy to apply, and the technical requirements are moderate (PC system, headphones and a two-pad response box). By changing picture-presentation time, or the correct-incorrect ratio, the difficulty of the paradigm can be adapted for special needs in patients with aphasia, motor dysfunctions, or low attentional status similar to so-called errorless learning [34], [35]. Of course, some cognitive impairments (e.g. severely decreased working memory) may impede successful learning [36].

Taken together, the paradigms put forward and tested here provide ample opportunity to further study the mechanisms underlying the acquisition of language skills in healthy and impaired subjects and provide an option to test the effects of innovative therapeutical strategies to enhance language reacquisition.

## Materials and Methods

### Rating of Stimulus Material

Photos of 100 everyday **actions** (such as eating, knitting, running), performed by 6 actors (3 female) in natural but differing environments were taken from different perspectives with an Olympus C5060 WZ digital camera. Pictures were achromatized. Text or distracting items were removed without compromising the graphic quality. Pictures were cut to position the action in the centre. Resolution was set to 72dpi. For each action, four different pictures with different actors and perspectives were selected.

Action-word pictures were then rated by a group of 28 students (age 23.1 years (y)±3.1 y; range 20–35 y; four male) from Muenster University, Germany. They were asked to write down the most appropriate German action word (verb) and to rate the pictures on a scale from 1 to 7 regarding a) the correctness of the suggested action word for the action, b) quality of depiction, c) motion association (general extent of movement) and d) involvement of particular body parts (“motor index” of head/face/mouth; arm/hand; leg/foot; whole body), e) daily-life frequency and f) frequency of personal performance of the action. A subset of action pictures was re-rated by 23 students at the University of Hamburg (age 26.8 y±7 y; range 21–58 y; four males) regarding a) the most appropriate German action word, b) recognisability of the action, c) motion association and motor indices, each on a scale from 1 to 7. For object-word learning, four different pictures were selected for each of 84 everyday **objects** (e.g. house, television, sun), either taken from the internet (www.wikipedia.com or www.wikisource.com) using GNU Free Documentation Licence (http://de.wikipedia.org/wiki/Wikipedia: GNU_Free_Documentation_License) or, if not freely available, with a Nikon D200 camera. Pictures were achromatized and cut to centre the object. Special care was taken not to include any distracting features.

All object pictures were rated by 22 students (age 27±7 y; range 21–58 y; six males) from Hamburg University, Germany. They were asked to write down the corresponding German object word and then rate the pictures on a scale from 1 to 7 for a) recognisability of the object, b) extent of movement in general and c) the association with particular body parts (head/face/mouth; arms/hands; legs; whole body).

All **novel words** (legal pseudowords such as digu, lare) were taken from an existing language-learning paradigm [15]. They were spoken by a female scientist, normalized with Cool-Edit® software to 70dB, high-pass (11025 Hz) and low-pass (50 Hz) filtered, and corrected for onset. The pseudowords were subsequently processed with the software Praat® and stored as.wav-files. Their mean duration was 970.3 ms±127.4 ms (±1SD). Pseudowords had previously been rated by 40 native German speakers, showed few associations with existing words and were of neutral emotional valence (see [15]). Studies investigating the acquisition of real languages (e.g. [37]) are hampered by the fact that multiple aspects of language acquisition (e.g. morphology, syntax, semantics and phonology) are naturally confounded, and face the difficulty of earlier exposure. We therefore opted for pseudowords, to have control over word length, difficulty of perception and phonological differences.

### Language-learning Paradigms

**Figure 2:**
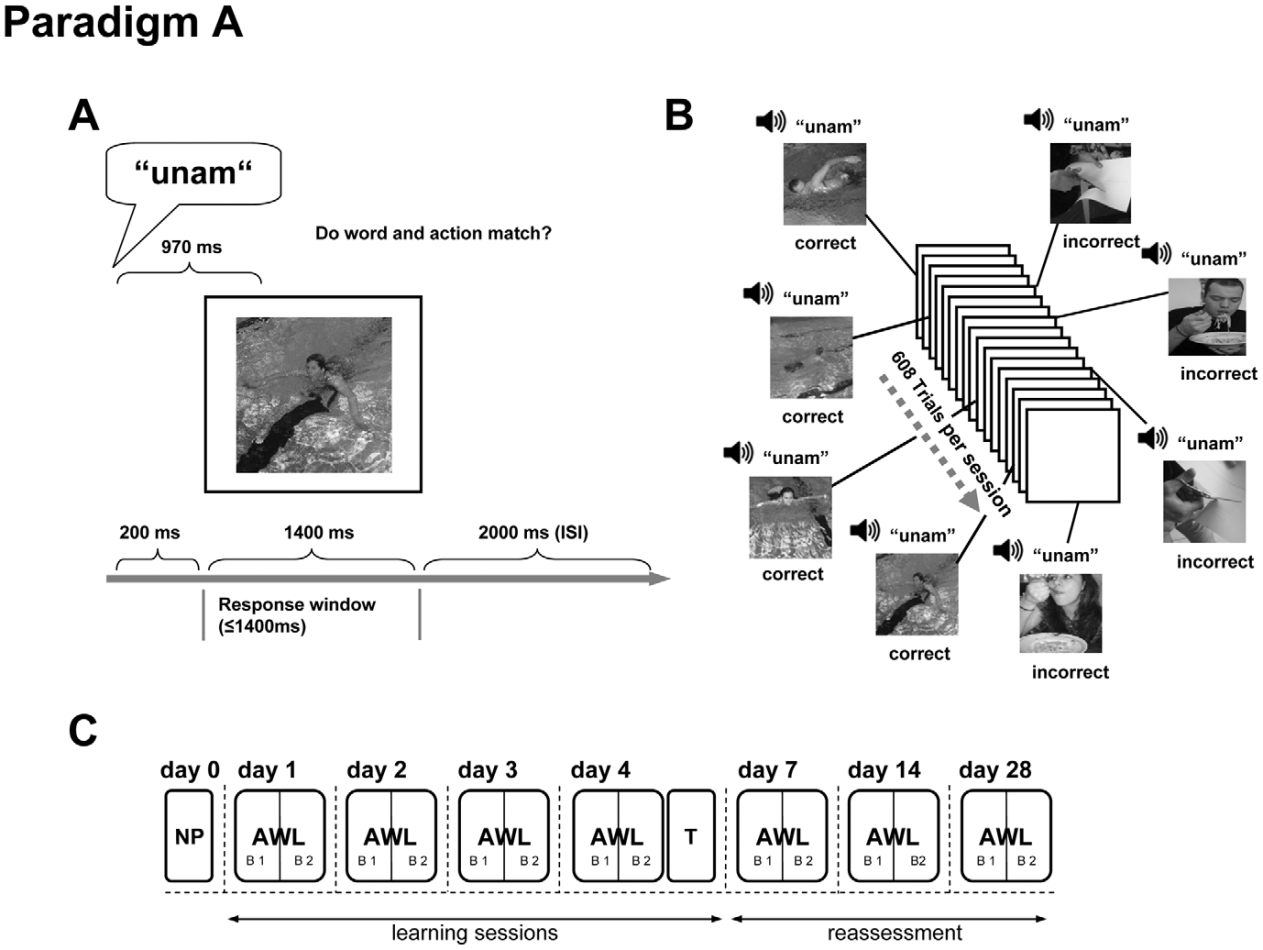
Design of Paradigm A. A Paradigm A single trial layout. Each trial is composed of one pseudoword in connection with a picture. 200 ms after onset of sound, the picture is shown and remains on the screen for 1400 ms representing the response window. After each stimulus pair is a pause of 2000 ms. B Paradigm A trial sequence during a learning session. Pseudowords are coupled with different pictures of actions. Correct couplings appear more often than incorrect couplings (for details see text). C Timeline of Paradigm A Four learning sessions are followed by the translation test and three reassessment sessions. NP: neuropsychological evaluation AWL: action word learning T: translation B: BlockISI: Interstimulus Interval.

Each selected action and object was coupled with pseudowords, presented repeatedly as “correct” or “incorrect” pairs, using the following schemes: For Paradigm A, 76 actions (depicted by four different photos) were each randomly assigned to a pseudoword (“correct” coupling) (Fig 2A). During one learning session, each individual photo was presented once with the correct pseudoword, and once with an incorrect one. Each action was thus presented eight times, four times with the “correct” pseudoword, and twice with two different “incorrect” pseudowords (4∶2 correct-incorrect ratio) (Fig 2B). A session with 76×8 = 608 trials was divided into two blocks of 304 trials each (Fig 2C). After each session, one of the two incorrect pseudowords was replaced by another incorrect one, while the correct coupling remained unchanged. This procedure ensured that each pseudoword and each picture was shown with the same frequency.

**Figure 3:**
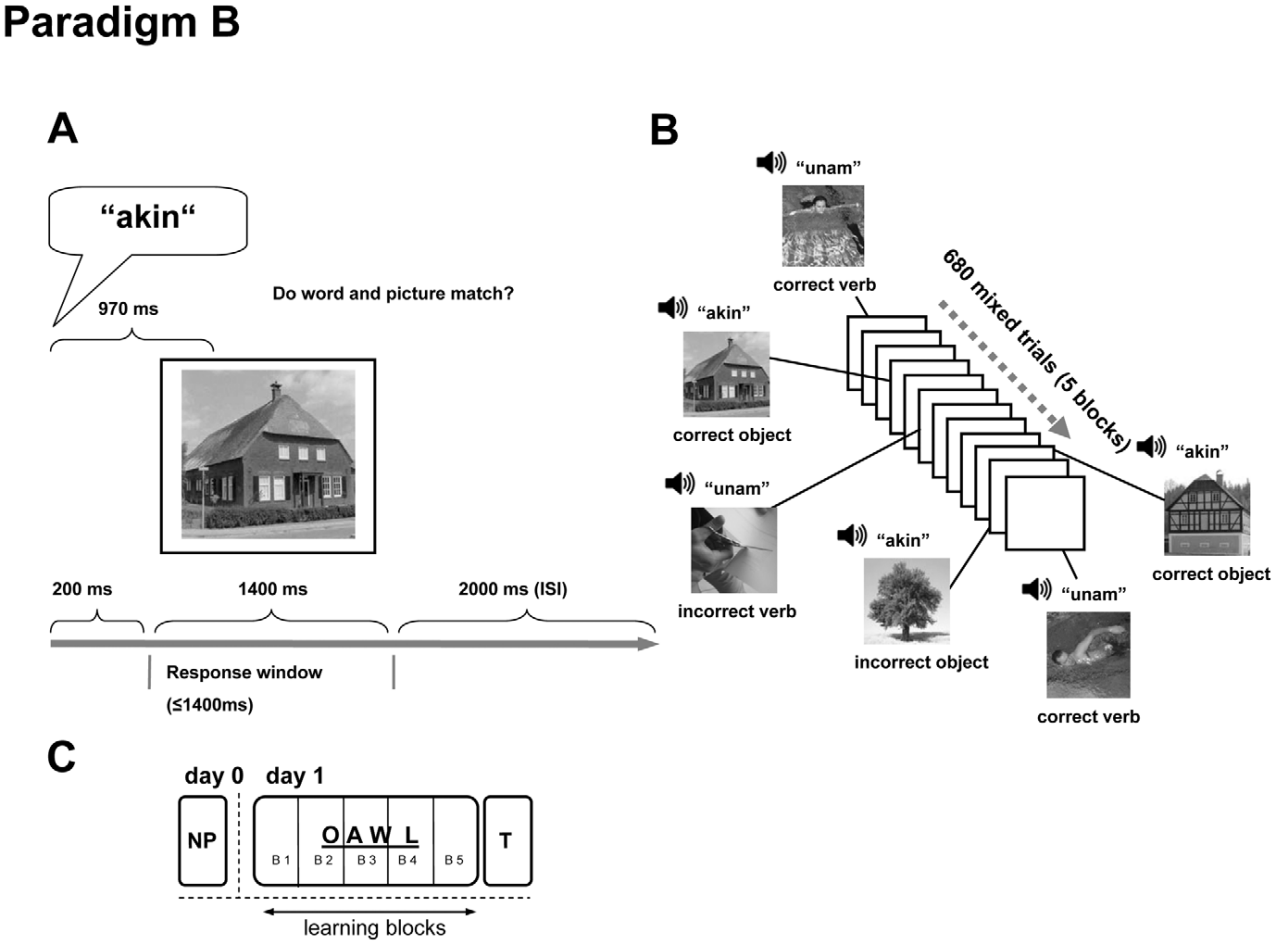
Design of Paradigm B. A Paradigm B single trial layout. Like in Paradigm A each trial is composed of one pseudoword in connection with a picture. Important change in Paradigm B is the occurrence of objects intermixed with actions (50% objects). B Paradigm B trial sequence during a learning session. Pseudowords are coupled with different pictures of actions and objects. Correct couplings appear more often than incorrect couplings (for details see text). C Timeline of Paradigm B Paradigm B consists of one single session, divided into five blocks and followed by the translation test. NP: neuropsychological evaluation OAWL: object and action word learning T: translation B: Block ISI: Interstimulus Interval.

For Paradigm B (Fig 3A), 17 objects and 17 actions, each represented by two different photos, were assigned to one of 34 pseudowords (“correct” coupling) (Fig 3A). During learning, the correct coupling was presented ten times, whereas each object and action was also presented once with a total of ten different pseudowords (“incorrect” couplings, correct-incorrect ratio 10∶1) (Fig 3B). This resulted in a total of 680 trials for the single-session training, divided into 5 blocks of 136 trials each. There was a pause of 2 min between blocks.

The order of trials was pseudorandomized in both paradigms, so that the same action, object, pseudoword or type of coupling (correct or incorrect) would appear maximally three times consecutively. Moreover, in Paradigm B, the same stimulus class (object or action) did not appear more than three times in a row.

In both paradigms, at each trial, subjects had to decide intuitively whether picture and pseudowords matched (“correct coupling”) or not (“incorrect coupling”). To learn a correct coupling, participants had to extract the correct action-meaning, and in paradigm B also object-meaning, from the different pictures, and to link this meaning to a pseudoword based on statistical probabilities.

### Subjects

All subjects gave written informed consent to participate in the study protocol. The study protocol was approved by the local ethics committee of the “Ärztekammer Hamburg” and was in accord with The Code of Ethics of the World Medical Association (Declaration of Helsinki; http://www.wma.net/e/policy/b3.htm). All subjects were naive to the experimental purpose of the study.

19 subjects participated (aged 25±0.7 y; Range 21–34 y; 8 male; all right handed except for one ambidextrous (Oldfield Handedness Score, [38])) in Paradigm A (a part of their data was already published in Liuzzi et al., 2010). For Paradigm B, 10 subjects (aged 26.2±0.74; range 24–32; 5 male; all right-handed except for two ambidextrous (Oldfield Handedness Score)) were tested. They were all monolingual native German speakers and students at the University of Hamburg. Exclusion criteria were participation in any of the ratings, bilingualism, a history of serious medical, neurological or psychiatric illnesses, and use of illegal, neuroactive (e.g. antidepressants, anticonvulsants etc.) or recreational drugs (including >15 cigarettes/day, >6 cups of coffee/day, >50 g of alcohol/day), as probed by a standardized questionnaire. Subjects who had a score above 14 in the Beck’s Depression Inventory [39] were also excluded.

Before language learning, participants were screened with an established neuropsychological test battery: verbal learning ability (VLMT: verbal learning and memory test; [40]), verbal fluency (Regensburg verbal fluency test: formal and semantic subtest, [41]), visuo-spatial memory and executive abilities (Rey-Osterrieth Complex Figure Test; [42]), attention (d2-Test, [43]) working memory (digit spans) and logical reasoning (Horn Intelligence test, subtest 4 [44]).

### Procedure

#### Learning Sessions

Subjects were seated in a comfortable armchair. They were instructed to decide intuitively whether action (in Paradigm B; action or object) and pseudoword matched (pressing the left mouse button with the right index finger) or not (pressing the right mouse button with the right middle finger). They were also told that they had to respond before the picture disappeared (1400 ms). Responses after time-out were scored as error. No feedback about the correctness of the response was provided during learning.

During training, photos of 5.4 cm^2^ size were presented at eye level on a 17 inch flat-screen monitor, with an approximate viewing distance of 75 cm. Onset of pseudowords was 200 ms prior to photo onset. On all trials, reaction times were recorded from picture onset until response or disappearance of the picture (1400 ms). The inter-trial interval was 2 s. The sound level was adjusted individually.

For Paradigm A, participants learning success was assessed during training on four consecutive days (LS 1–4), and after 7, 14 and 28 days (reassessment sessions RS 1–3) (Fig 2C). Each training session lasted approximately 45 min. The learning session of day 1 was repeated in the reassessment sessions. Moreover, at the end of day 4, subjects translated the 76 written novel action words into German (“translations”).

For Paradigm B, learning success was assessed during a single session, divided into 5 blocks, which lasted approximately one hour (Fig 3C). After the training session, all 34 pseudowords were presented twice (with a 2 s inter-trial interval) without pictures. Participants were asked to translate the pseudowords into German.

To further substantiate whether learning action-related words differs as a function of motor association, we analyzed two sets of action-related words from Paradigm A, which differed according to transitivity and motor indices.

#### Data Analysis

The first measure for successful learning in both paradigms was the percentage of correct answers in the translation test (translation of pseudowords into the participantś native (German) language). We additionally analysed learning success (i.e. the percentage of correct answers per session/block) over time. For Paradigm A, the percentage of correct decisions during training and reaction times were analysed using a repeated-measures analysis of variance (rmANOVA) involving the factor “time” (7 levels: days 1, 2, 3, 4, 7, 14, 28).

For Paradigm B, we performed a two-way rmANOVA on correct decisions and reaction times with the factors “time” (5 levels: Block 1–5) and “word category” (two levels: “action word” and “object word”). The difference of delayed or no responses between the two word categories was assessed by a two-way rmANOVA involving the factors “time” and “word category”. A paired two-tailed t-test was used to assess differences between the translation rates for action- and object-related words (Paradigm B).

In a sub-analysis, we defined two groups of action-related words with regard to transitivity (according to the lexical database by the University of Leipzig “Wortschatz”). Using two-tailed t-tests for independent samples, we compared these two groups of action-related words with respect to motion association and motor indices, and to potential confounders such as naming consistency, lexical word frequency, quality of depiction and distractors, as obtained in the above mentioned rating study. Two-sample t-tests between transitive and intransitive action-related words were calculated for the percentage of correct answers in the translation test. In a next step, we calculated a rmANOVA with the factors “time” (7 levels: LS1–LS4, RS1–RS3) and “transitivity” (2 levels: transitive, intransitive) for associative learning results.

Kolmogorov-Smirnov-tests for normal distribution were calculated before statistical parametric testing. All ANOVA results were Greenhouse-Geisser corrected if assumptions of sphericity were violated. Bonferroni corrected t-tests were used for all post-hoc tests. Corrected p values are given in the results section. Results were considered significant at a level of p<0.05. All data are expressed as mean ± standard error unless stated differently. Statistical analyses were done using SPSS 15.0 ®.

## Supporting Information

Table S1 **Rating for Paradigm A.** This is a list of all body related actions rated for Paradigm A. On the questionnaire there were 8 questions: 1 Please name the given action. 2 Please rate its appropriateness. 3 Please rate its quality of depiction from 1–7. (1 being best) 4 Please name distractors if applicable. 5 How strong is the depicted item associated with motion? (7 being most) 6 How strong are different body parts (arm/hand, leg/foot, head and whole body) associated with the object? (7 being most) 7 How often does this action occur in daily life? (1 seldom, 7 often) 8 How often do you perform this action? (1 seldom, 7 often)
(DOC)

Table S2 **Rating for Paradigm B – Rating objects.** This is a list of all the objects rated for Paradigm B. On the questionnaire there were 4 items: 1 Please name the given item. 2 Please rate its recognizability from 1–7. (1 being best) 3 How strong is the depicted item associated with motion? (7 being most) 4 How strong are different body parts (arm/hand, leg/foot, head and whole body) associated with the object? (7 being most). Optionally raters could comment the pictures.
(DOC)

Table S3 **Rating for Paradigm B – Rating verbs.** This is a list of all the verbs rated for Paradigm B. On the questionnaire there were 4 items:1 Please name the given item. 2 Please rate its recognizability from 1–7. (1 being best) 3 How strong is the depicted item associated with motion? (7 being most) 4 How strong are different body parts (arm/hand, leg/foot, head and whole body) associated with the verb? (7 being most). Optionally raters could comment the pictures.
(DOC)

Table S4 **Results of neuropsychological testing.**
(DOC)

## References

[1] Pedersen PM, Jorgensen HS, Nakayama H, Raaschou HO, Olsen TS (1995). Aphasia in acute stroke: incidence, determinants, and recovery.. Ann Neurol.

[2] Kelly-Hayes M, Beiser A, Kase CS, Scaramucci A, D’Agostino RB (2003). The influence of gender and age on disability following ischemic stroke: the Framingham study.. J Stroke Cerebrovasc Dis.

[3] Schwartz RG, Leonard LB (1984). Words, objects, and actions in early lexical acquisition.. J Speech Hear Res.

[4] Pulvermuller F, Berthier ML (2008). Aphasia therapy on a neuroscience basis.. Aphasiology.

[5] Hebb DO (1949). The organization of behavior: A neurophysiological theory..

[6] Pulvermüller F (1999). Words in the brain’s language.. Behavioral and Brain Science.

[7] Nickels L (2002). Improving word finding: Practice makes (closer to) perfect?. Aphasiology.

[8] Pulvermuller F, Neininger B, Elbert T, Mohr B, Rockstroh B (2001). Constraint-induced therapy of chronic aphasia after stroke.. Stroke.

[9] Szaflarski JP, Ball A, Grether S, Al-Fwaress F, Griffith NM (2008). Constraint-induced aphasia therapy stimulates language recovery in patients with chronic aphasia after ischemic stroke.. Med Sci Monit.

[10] Liuzzi G, Ellger T, Floel A, Breitenstein C, Jansen A (2008). Walking the talk–speech activates the leg motor cortex.. Neuropsychologia.

[11] Rizzolatti G, Arbib MA (1998). Language within our grasp.. Trends Neurosci.

[12] Hauk O, Johnsrude I, Pulvermuller F (2004). Somatotopic representation of action words in human motor and premotor cortex.. Neuron.

[13] Pulvermuller F, Harle M, Hummel F (2000). Neurophysiological distinction of verb categories.. Neuroreport.

[14] Pulvermuller F, Harle M, Hummel F (2001). Walking or talking? Behavioral and neurophysiological correlates of action verb processing.. Brain Lang.

[15] Breitenstein C, Knecht S (2002). Development and validation of a language learning model for behavioral and functional-imaging studies.. J Neurosci Methods.

[16] Dobel C, Junghofer M, Breitenstein C, Klauke B, Knecht S (2010). New names for known things: on the association of novel word forms with existing semantic information.. J Cogn Neurosci.

[17] de Vries MH, Barth AC, Maiworm S, Knecht S, Zwitserlood P (2010). Electrical stimulation of Broca’s area enhances implicit learning of an artificial grammar.. J Cogn Neurosci.

[18] Breitenstein C, Jansen A, Deppe M, Foerster AF, Sommer J (2005). Hippocampus activity differentiates good from poor learners of a novel lexicon.. Neuroimage.

[19] Floel A, Rosser N, Michka O, Knecht S, Breitenstein C (2008). Noninvasive Brain Stimulation Improves Language Learning..

[20] Knecht S, Breitenstein C, Bushuven S, Wailke S, Kamping S (2004). Levodopa: faster and better word learning in normal humans.. Annals of Neurology.

[21] Liuzzi G, Freundlieb N, Ridder V, Hoppe J, Heise K (2010). The involvement of the left motor cortex in learning of a novel action word lexicon.. Curr Biol.

[22] Enticott PG, Kennedy HA, Bradshaw JL, Rinehart NJ, Fitzgerald PB (2010). Understanding mirror neurons: evidence for enhanced corticospinal excitability during the observation of transitive but not intransitive hand gestures.. Neuropsychologia.

[23] Knecht S, Breitenstein C, Bushuven S, Wailke S, Kamping S (2004). Levodopa: faster and better word learning in normal humans.. Ann Neurol.

[24] Reber AS (1967). Implicit learning of artificial grammars.. J Verb Learn Verb Behav.

[25] Dienes Z (2008). Subjective measures of unconscious knowledge.. Prog Brain Res.

[26] Miller GA, Fellbaum C (1991). Semantic networks of English.. Cognition.

[27] Matzig S, Druks J, Masterson J, Vigliocco G (2009). Noun and verb differences in picture naming: past studies and new evidence.. Cortex.

[28] Bird H, Howard D, Franklin S (2000). Why is a verb like an inanimate object? Grammatical category and semantic category deficits.. Brain Lang.

[29] Pulvermuller F (2005). Brain mechanisms linking language and action.. Nat Rev Neurosci.

[30] Breitenstein C, Kamping S, Jansen A, Schomacher M, Knecht S (2004). Word learning can be achieved without feedback: implications for aphasia therapy.. Restor Neurol Neurosci.

[31] Hummel FC, Cohen LG (2006). Non-invasive brain stimulation: a new strategy to improve neurorehabilitation after stroke?. Lancet Neurol.

[32] Floel A, Cohen LG (2010). Recovery of function in humans: cortical stimulation and pharmacological treatments after stroke.. Neurobiol Dis.

[33] Berthier ML, Green C, Lara JP, Higueras C, Barbancho MA (2009). Memantine and constraint-induced aphasia therapy in chronic poststroke aphasia.. Ann Neurol.

[34] Conroy P, Sage K, Lambon RMA (2009). Errorless and errorful therapy for verb and noun naming in aphasia.. Aphasiology.

[35] Fillingham JK, Hodgson C, Sage K, Ralph MAL (2003). The application of errorless learning to aphasic disorders: A review of theory and practice.. Neuropsychological Rehabilitation: An International Journal.

[36] Breitenstein C, Kramer K, Meinzer M, Baumgartner A, Floel A (2009). [Intense language training for aphasia. Contribution of cognitive factors].. Nervenarzt 80: 149–150,.

[37] Musso M, Moro A, Glauche V, Rijntjes M, Reichenbach J (2003). Broca’s area and the language instinct.. Nat Neurosci.

[38] Oldfield RC (1971). The assessment and analysis of handedness: the Edinburgh inventory.. Neuropsychologia.

[39] Beck AT (1995). Beck-Depressions-Inventar..

[40] Helmstaedter C, Lendt M, Lux S (2001). Verbaler Lern- und Merkfähigkeitstest..

[41] Aschenbrenner S, Tucha O, Lange KW (2000). Regensburger Wortflüssigkeits-Test..

[42] Rey A (1959). Manuel du test de copie d’une figure complexe de A. Rey..

[43] Brickenkamp R (2002). Test d2 - Aufmerksamkeits-Belastungs-Test..

[44] Horn W (1983). Leistungsprüfsystem..

