# Supplementary material for: Associative Vocabulary Learning: Development and Testing of Two Paradigms for the (Re-) Acquisition of Action- and Object-Related Words
Source: PLoS One. 2012 Jun 6;7(6):e37033. doi: 10.1371/journal.pone.0037033 (PMC3368912; doi:10.1371/journal.pone.0037033)
Supplement: Table S1 — Rating for Paradigm A. This is a list of all body related actions rated for Paradigm A. On the questionnaire there were 8 questions: 1 Please name the given action. 2 Please rate its appropriateness. 3 Please rate its quality of depiction from 1–7. (1 being best) 4 Please name distractors if applicable. 5 How strong is the depicted item associated with motion? (7 being most) 6 How strong are different body parts (arm/hand, leg/foot, head and whole body) associated with the object? (7 being most) 7 How often does this action occur in daily life? (1 seldom, 7 often) 8 How often do you perform this action? (1 seldom, 7 often) (DOC) [file pone.0037033.s001.doc]

**Table S1 Rating for Paradigm A**

| action  (* used in Paradigm A) | sample picture | German verb | 1: naming consist-ency | 2: appropriat-ness | 3: quality of depiction | 4: dis-tractors (in %) | 5: associat-ion to motion | 6: association to | | | | daily frequency | own frequency |
| --- | --- | --- | --- | --- | --- | --- | --- | --- | --- | --- | --- | --- | --- |
|  |  |  |  |  |  |  |  | head | arm | leg | whole body |  |  |
| to auscul-tate | 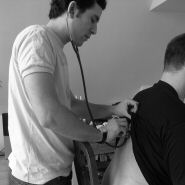 | abhoeren | 100,00 | 6,50  +/-0,94 | 2,00  +/-1,11 | 35,70 | 2,79  +/-1,53 | 1,71  +/-0,91 | 5,14  +/-1,51 | 1,00  +/-0,00 | 1,71  +/-1,38 | 2,57  +/-1,09 | 1,14  +/-0,53 |
| to dry | 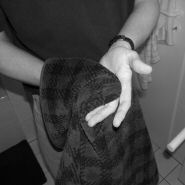 | abtrocknen | 7,70 | 3,31  +/-1,80 | 6,31  +/-1,18 | 69,20 | 2,77  +/-1,42 | 1,23  +/-0,60 | 5,00  +/-1,87 | 1,23  +/-0,44 | 1,38  +/-0,51 | 6,00  +/-1,29 | 5,46  +/-1,66 |
| to dress | 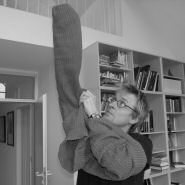 | anziehen | 89,30 | 6,54  +/-0,72 | 2,18  +/-1,62 | 10,70 | 4,21  +/-1,47 | 2,79  +/-1,66 | 5,86  +/-1,16 | 2,39  +/-1,66 | 3,71  +/-1,58 | 6,39  +/-0,89 | 6,43  +/-0,89 |
| to  hang up * | 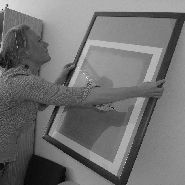 | aufhaengen | 100,00 | 6,61  +/-1,02 | 1,71  +/-1,07 | 7,15 | 4,00  +/-1,38 | 2,25  +/-1,61 | 5,96  +/-1,37 | 1,93  +/-1,12 | 3,39  +/-1,46 | 3,50  +/-1,21 | 2,71  +/-1,41 |
| to  blow out * | 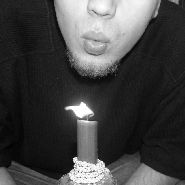 | auspusten | 64,30 | 6,07  +/-1,59 | 2,29  +/-1,68 | 14,30 | 2,29  +/-0,99 | 5,86  +/-1,66 | 1,29  +/-0,61 | 1,29  +/-0,61 | 1,43  +/-0,85 | 3,50  +/-1,40 | 2,71  +/-1,59 |
| to balance * | 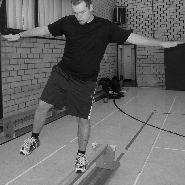 | balancieren | 89,30 | 6,75  +/-0,50 | 1,96  +/-1,20 | 25,00 | 5,52  +/-1,05 | 2,75  +/-1,93 | 6,00  +/-1,16 | 5,86  +/-1,31 | 6,29  +/-0,84 | 2,79  +/-1,23 | 2,50  +/-1,51 |
| to pray * | 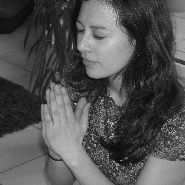 | beten | 92,90 | 6,79  +/-0,58 | 2,93  +/-1,64 | 35,70 | 1,93  +/-1,44 | 3,93  +/-1,98 | 5,43  +/-1,91 | 1,21  +/-0,80 | 1,86  +/-1,35 | 4,29  +/-1,26 | 2,57  +/-1,87 |
| to bind * | 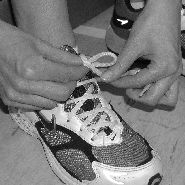 | binden | 100,00 | 6,64  +/-0,50 | 1,93  +/-1,82 | 0,00 | 2,71  +/-1,14 | 1,71  +/-1,27 | 5,71  +/-1,49 | 2,14  +/-2,14 | 1,86  +/-1,10 | 5,64  +/-1,08 | 5,14  +/-2,03 |
| to box * | 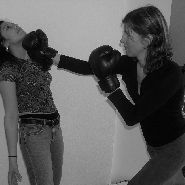 | boxen | 100,00 | 6,86  +/-0,36 | 1,39  +/-0,98 | 3,55 | 6,04  +/-0,79 | 3,36  +/-1,91 | 6,75  +/-0,46 | 4,89  +/-1,69 | 5,50  +/-1,63 | 1,89  +/-0,84 | 1,29  +/-0,95 |
| to iron * | 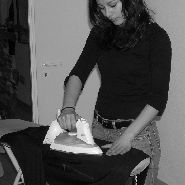 | buegeln | 100,00 | 6,93  +/-0,27 | 1,50  +/-1,61 | 0,00 | 3,23  +/-1,24 | 2,00  +/-1,36 | 5,86  +/-1,29 | 1,71  +/-0,99 | 2,57  +/-1,50 | 4,29  +/-0,91 | 2,57  +/-1,22 |
| to stretch * | 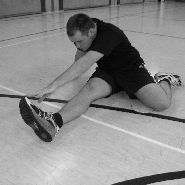 | dehnen | 92,80 | 6,50  +/-0,65 | 1,71  +/-1,38 | 0,00 | 4,21  +/-1,31 | 1,93  +/-1,44 | 5,79  +/-0,97 | 5,50  +/-1,34 | 5,64  +/-1,74 | 3,43  +/-1,22 | 2,86  +/-1,70 |
| to think * | 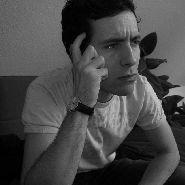 | denken | 62,82 | 5,41  +/-1,52 | 3,34  +/-1,67 | 30,00 | 1,94  +/-1,09 | 4,51  +/-2,21 | 2,90  +/-1,86 | 1,03  +/-0,11 | 1,29  +/-0,70 | 6,44  +/-0,87 | 6,33  +/-0,99 |
| to threaten * | 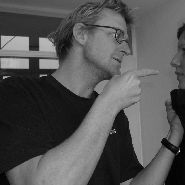 | drohen | 43,40 | 5,36  +/-1,47 | 3,30  +/-1,75 | 22,53 | 3,27  +/-1,24 | 4,92  +/-1,97 | 5,07  +/-1,63 | 1,53  +/-0,98 | 2,84  +/-1,53 | 3,14  +/-1,22 | 1,58  +/-0,68 |
| to push | 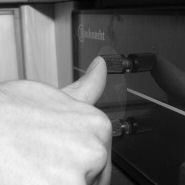 | druecken | 57,10 | 5,71  +/-1,54 | 3,14  +/-1,99 | 42,90 | 2,14  +/-0,95 | 1,50  +/-0,94 | 5,64  +/-1,60 | 1,00  +/-0,00 | 1,14  +/-0,36 | 5,00  +/-1,18 | 4,93  +/-1,33 |
| to curl * | 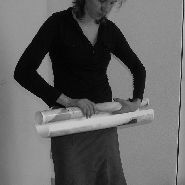 | einrollen | 100,00 | 6,57  +/-0,71 | 1,68  +/-1,16 | 7,10 | 2,75  +/-1,39 | 1,43  +/-1,09 | 5,64  +/-1,69 | 1,04  +/-0,14 | 1,39  +/-0,75 | 2,82  +/-1,28 | 2,11  +/-1,01 |
| to eat * | 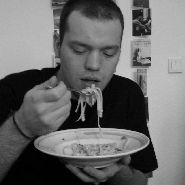 | essen | 100,00 | 6,93  +/-0,27 | 1,71  +/-1,68 | 21,40 | 2,57  +/-1,09 | 5,71  +/-1,64 | 5,43  +/-1,70 | 1,00  +/-0,00 | 1,43  +/-0,85 | 6,50  +/-0,85 | 6,57  +/-0,65 |
| to ride * | 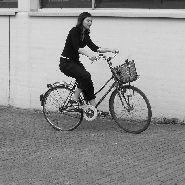 | fahren | 100,00 | 5,93  +/-1,77 | 2,50  +/-2,21 | 21,40 | 5,00  +/-1,18 | 2,00  +/-1,80 | 5,21  +/-1,58 | 6,14  +/-0,86 | 5,00  +/-1,84 | 5,57  +/-0,85 | 5,36  +/-2,13 |
| to fall * | 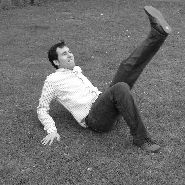 | fallen | 71,45 | 5,86  +/-1,06 | 3,11  +/-1,59 | 25,00 | 5,32  +/-1,37 | 3,00  +/-1,73 | 5,14  +/-1,87 | 5,64  +/-1,19 | 6,32  +/-1,08 | 3,14  +/-1,15 | 2,11  +/-1,24 |
| to catch | 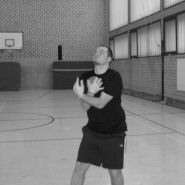 | fangen | 64,30 | 5,43  +/-1,34 | 3,71  +/-2,05 | 57,10 | 4,07  +/-1,21 | 2,14  +/-1,66 | 6,31  +/-1,11 | 2,50  +/-1,87 | 3,50  +/-1,51 | 3,50  +/-1,16 | 3,00  +/-1,62 |
| to fence * | 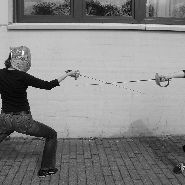 | fechten | 96,45 | 6,79  +/-0,68 | 1,61  +/-1,60 | 0,00 | 5,82  +/-0,83 | 2,75  +/-1,81 | 6,36  +/-0,97 | 5,96  +/-1,00 | 6,18  +/-0,81 | 1,64  +/-0,62 | 1,00  +/-0,00 |
| to tie up | 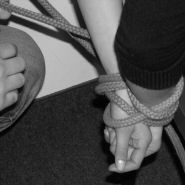 | fesseln | 50,00 | 6,33  +/-1,23 | 2,33  +/-1,87 | 33,30 | 2,83  +/-0,72 | 1,67  +/-1,37 | 5,67  +/-1,61 | 1,58  +/-1,44 | 1,83  +/-1,40 | 1,33  +/-0,49 | 1,08  +/-0,29 |
| to film * | 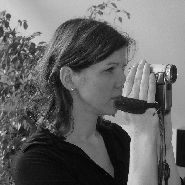 | filmen | 100,00 | 6,43  +/-0,94 | 1,71  +/-1,27 | 7,10 | 2,07  +/-1,07 | 3,71  +/-2,23 | 4,79  +/-2,15 | 1,57  +/-1,02 | 1,64  +/-1,08 | 2,71  +/-1,14 | 1,43  +/-0,76 |
| to whisper * | 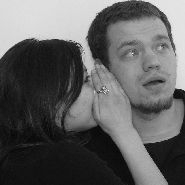 | fluestern | 96,45 | 6,82  +/-0,37 | 1,61  +/-1,47 | 3,55 | 2,25  +/-1,07 | 5,46  +/-1,86 | 4,36  +/-1,80 | 1,39  +/-1,47 | 1,61  +/-0,91 | 4,18  +/-1,50 | 3,21  +/-1,61 |
| to take a photo * | 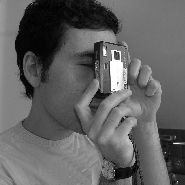 | fotografieren | 100,00 | 6,86  +/-0,36 | 1,86  +/-1,66 | 21,40 | 2,43  +/-0,85 | 4,21  +/-2,29 | 4,71  +/-2,02 | 1,29  +/-0,83 | 1,36  +/-0,93 | 4,00  +/-0,88 | 3,14  +/-1,83 |
| to freeze * | 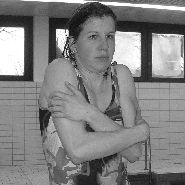 | frieren | 85,70 | 5,86  +/-1,83 | 1,86  +/-0,95 | 38,50 | 4,43  +/-1,40 | 3,79  +/-2,04 | 5,21  +/-1,63 | 3,21  +/-1,67 | 5,14  +/-1,56 | 4,36  +/-1,28 | 3,86  +/-1,46 |
| to yawn * | 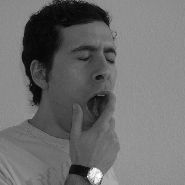 | gaehnen | 92,90 | 6,64  +/-0,98 | 1,57  +/-1,63 | 14,30 | 3,00  +/-1,24 | 5,96  +/-1,46 | 4,25  +/-1,66 | 1,04  +/-0,14 | 1,46  +/-0,74 | 5,14  +/-1,39 | 5,11  +/-1,49 |
| to walk * | 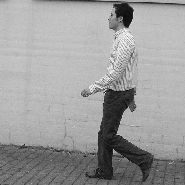 | gehen | 100,00 | 6,71  +/-0,51 | 1,96  +/-1,48 | 14,30 | 5,09  +/-1,30 | 1,71  +/-0,84 | 3,79  +/-1,75 | 6,32  +/-0,80 | 5,57  +/-1,13 | 6,89  +/-0,32 | 6,93  +/-0,18 |
| to hammer | 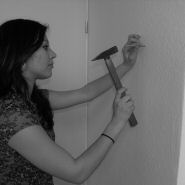 | haemmern | 92,80 | 6,00  +/-1,52 | 2,29  +/-1,86 | 7,10 | 3,29  +/-0,73 | 2,00  +/-1,75 | 5,93  +/-1,49 | 1,36  +/-0,63 | 1,79  +/-0,80 | 3,21  +/-1,19 | 2,29  +/-1,44 |
| to lift | 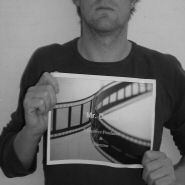 | heben | 92,90 | 6,07  +/-0,92 | 2,64  +/-1,74 | 14,30 | 3,86  +/-1,10 | 1,79  +/-1,42 | 5,93  +/-0,92 | 3,00  +/-1,75 | 4,29  +/-1,38 | 4,64  +/-0,93 | 4,43  +/-0,94 |
| to hear * | 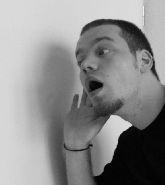 | hoeren | 100,00 | 6,36  +/-0,51 | 2,00  +/-0,75 | 14,30 | 1,71  +/-1,07 | 4,64  +/-1,98 | 2,21  +/-1,85 | 1,00  +/-0,00 | 1,14  +/-0,53 | 6,43  +/-0,94 | 6,36  +/-0,93 |
| to cough * | 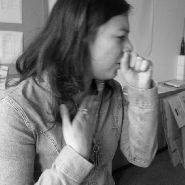 | husten | 100,00 | 6,86  +/-0,36 | 1,50  +/-1,34 | 28,60 | 3,64  +/-1,78 | 5,79  +/-1,76 | 5,00  +/-2,25 | 1,00  +/-0,00 | 2,64  +/-1,95 | 4,86  +/-1,41 | 3,50  +/-1,02 |
| to juggle | 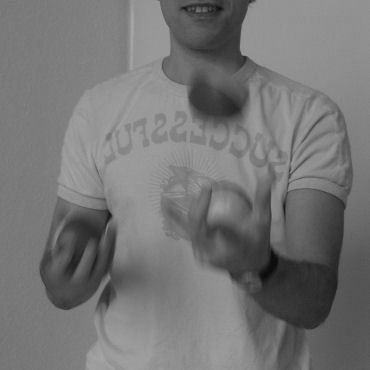 | jonglieren | 71,40 | 6,57  +/-0,85 | 2,07  +/-1,69 | 21,40 | 3,93  +/-1,21 | 2,79  +/-1,89 | 6,21  +/-1,12 | 1,93  +/-1,33 | 2,43  +/-0,85 | 1,57  +/-0,51 | 1,07  +/-0,27 |
| to cheer * | 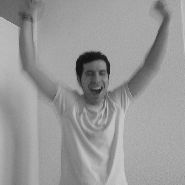 | jubeln | 88,13 | 6,26  +/-1,37 | 2,10  +/-1,67 | 14,27 | 4,83  +/-1,57 | 5,17  +/-1,79 | 5,93  +/-1,27 | 2,29  +/-1,25 | 4,14  +/-1,56 | 4,07  +/-1,26 | 3,33  +/-1,32 |
| to clap * | 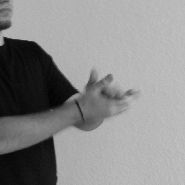 | klatschen | 78,50 | 6,21  +/-0,97 | 3,57  +/-1,91 | 35,70 | 4,36  +/-1,50 | 3,00  +/-2,08 | 6,64  +/-0,63 | 1,36  +/-0,50 | 2,50  +/-1,40 | 4,00  +/-1,04 | 3,43  +/-0,94 |
| to climb * | 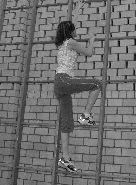 | klettern | 100,00 | 6,96  +/-0,14 | 1,86  +/-2,41 | 0,00 | 6,25  +/-0,59 | 2,32  +/-1,87 | 6,27  +/-0,81 | 6,43  +/-0,66 | 6,43  +/-0,78 | 2,64  +/-1,06 | 2,11  +/-1,15 |
| to knock | 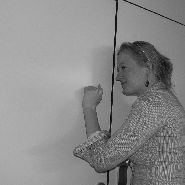 | klopfen | 100,00 | 6,93  +/-0,27 | 1,57  +/-0,76 | 7,10 | 3,29  +/-1,54 | 1,64  +/-1,22 | 6,00  +/-1,36 | 1,00  +/-0,00 | 1,64  +/-1,08 | 4,57  +/-1,50 | 4,57  +/-1,45 |
| to pinch* | 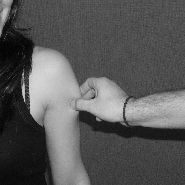 | kneifen | 100,00 | 6,79  +/-0,43 | 1,64  +/-1,65 | 0,00 | 2,07  +/-0,62 | 1,50  +/-0,76 | 5,50  +/-1,56 | 1,00  +/-0,00 | 1,14  +/-0,36 | 2,00  +/-0,78 | 1,43  +/-0,65 |
| to cook | 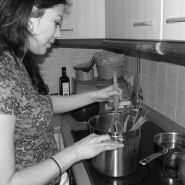 | kochen | 75,00 | 6,68  +/-0,73 | 1,93  +/-1,05 | 39,30 | 2,96  +/-1,22 | 1,82  +/-1,36 | 5,25  +/-1,75 | 1,54  +/-0,85 | 2,07  +/-1,21 | 5,89  +/-1,02 | 4,86  +/-1,57 |
| to vomit * | 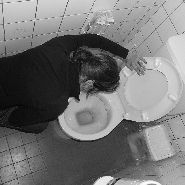 | kotzen | 85,70 | 6,29  +/-1,44 | 2,00  +/-1,52 | 0,00 | 4,43  +/-1,55 | 6,31  +/-0,95 | 4,00  +/-2,00 | 1,71  +/-0,91 | 4,36  +/-1,91 | 2,86  +/-1,41 | 1,43  +/-0,51 |
| to kiss * | 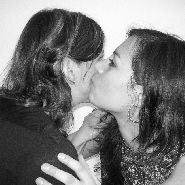 | kuessen | 100,00 | 6,86  +/-0,36 | 1,86  +/-1,66 | 14,30 | 2,57  +/-1,09 | 5,79  +/-1,76 | 2,36  +/-1,34 | 1,00  +/-0,00 | 1,43  +/-0,65 | 4,86  +/-0,77 | 3,93  +/-1,86 |
| to laugh * | 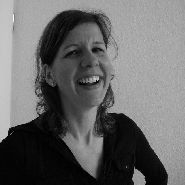 | lachen | 96,45 | 6,79  +/-0,57 | 1,57  +/-1,12 | 7,15 | 2,50  +/-1,12 | 6,21  +/-1,15 | 1,93  +/-1,14 | 1,11  +/-0,29 | 2,54  +/-1,57 | 5,71  +/-1,24 | 5,64  +/-1,19 |
| to varnish | 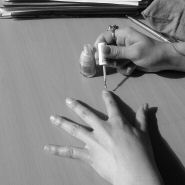 | lackieren | 78,60 | 6,79  +/-0,43 | 1,64  +/-1,60 | 7,10 | 2,07  +/-0,83 | 1,79  +/-1,37 | 5,29  +/-1,73 | 1,00  +/-0,00 | 1,07  +/-0,27 | 3,64  +/-1,22 | 2,00  +/-1,41 |
| to read * | 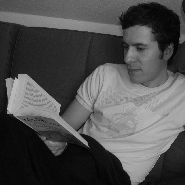 | lesen | 100,00 | 6,93  +/-0,27 | 1,43  +/-1,60 | 0,00 | 1,64  +/-0,84 | 3,86  +/-2,51 | 2,93  +/-1,64 | 1,00  +/-0,00 | 1,21  +/-0,43 | 5,21  +/-1,12 | 5,93  +/-1,21 |
| to perforate * | 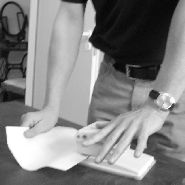 | lochen | 92,90 | 6,86  +/-0,36 | 1,50  +/-1,61 | 14,30 | 2,00  +/-0,55 | 1,43  +/-1,09 | 4,86  +/-1,92 | 1,00  +/-0,00 | 1,23  +/-0,60 | 3,21  +/-1,12 | 3,36  +/-1,69 |
| to massage * | 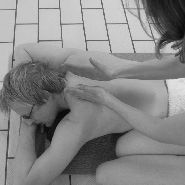 | massieren | 89,30 | 6,50  +/-0,76 | 2,32  +/-1,43 | 17,85 | 3,93  +/-1,23 | 1,54  +/-1,34 | 6,04  +/-1,39 | 1,36  +/-0,62 | 2,29  +/-1,37 | 3,11  +/-0,75 | 2,11  +/-1,30 |
| to signal in class * | 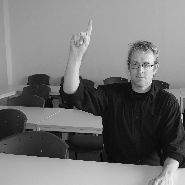 | melden | 92,90 | 6,48  +/-1,27 | 1,88  +/-1,54 | 4,73 | 2,83  +/-1,31 | 2,29  +/-1,46 | 5,48  +/-1,54 | 1,07  +/-0,19 | 1,62  +/-0,73 | 3,74  +/-1,38 | 2,62  +/-1,41 |
| to sew * | 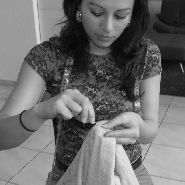 | naehen | 100,00 | 6,71  +/-0,61 | 2,21  +/-1,63 | 21,40 | 2,43  +/-0,76 | 1,79  +/-1,48 | 5,46  +/-1,51 | 1,07  +/-0,27 | 1,21  +/-0,58 | 3,29  +/-1,14 | 2,00  +/-1,11 |
| to open | 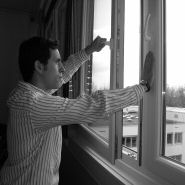 | oeffnen | 75,00 | 6,36  +/-0,78 | 2,54  +/-1,33 | 28,60 | 2,79  +/-1,07 | 1,50  +/-0,92 | 5,36  +/-1,71 | 1,04  +/-0,14 | 1,61  +/-0,62 | 5,71  +/-1,31 | 5,46  +/-1,36 |
| to pacl * | 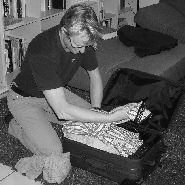 | packen | 96,45 | 6,71  +/-0,44 | 1,64  +/-1,01 | 10,70 | 3,32  +/-1,22 | 1,46  +/-0,79 | 5,32  +/-1,57 | 1,89  +/-1,26 | 2,57  +/-1,37 | 4,00  +/-1,22 | 4,25  +/-1,60 |
| to whistle * | 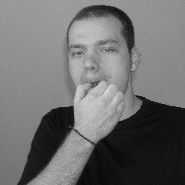 | pfeifen | 100,00 | 6,89  +/-0,22 | 1,43  +/-0,82 | 7,15 | 2,86  +/-1,55 | 5,93  +/-1,37 | 5,18  +/-1,66 | 1,04  +/-0,14 | 1,50  +/-0,94 | 3,39  +/-1,28 | 1,75  +/-1,02 |
| to pump * | 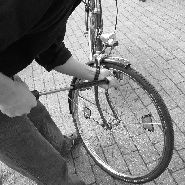 | pumpen | 100,00 | 6,79  +/-0,43 | 1,64  +/-1,34 | 0,00 | 3,29  +/-1,07 | 1,71  +/-1,07 | 5,71  +/-1,64 | 1,36  +/-0,63 | 2,29  +/-1,27 | 3,21  +/-1,05 | 2,21  +/-1,25 |
| to puzzle * | 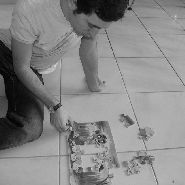 | puzzlen | 92,85 | 6,75  +/-0,83 | 1,50  +/-1,24 | 3,55 | 2,61  +/-0,99 | 2,36  +/-1,71 | 4,93  +/-1,69 | 1,04  +/-0,14 | 1,75  +/-0,90 | 2,54  +/-1,11 | 1,25  +/-0,50 |
| to erase | 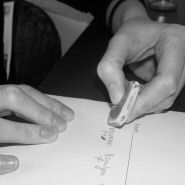 | radieren | 71,40 | 6,00  +/-1,41 | 2,29  +/-1,07 | 14,30 | 2,14  +/-0,77 | 1,54  +/-0,97 | 4,69  +/-2,02 | 1,00  +/-0,00 | 1,15  +/-0,38 | 3,69  +/-1,32 | 3,46  +/-1,71 |
| to shave * | 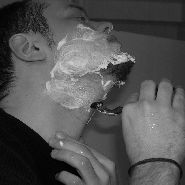 | rasieren | 84,60 | 6,77  +/-0,60 | 2,77  +/-2,28 | 46,20 | 2,69  +/-0,95 | 4,69  +/-2,18 | 4,92  +/-2,18 | 1,00  +/-0,00 | 1,15  +/-0,55 | 4,85  +/-0,80 | 4,00  +/-1,96 |
| to smoke * | 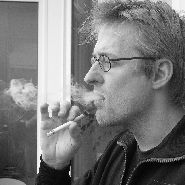 | rauchen | 100,00 | 6,92  +/-0,28 | 1,77  +/-1,69 | 7,70 | 1,85  +/-0,69 | 4,46  +/-2,63 | 4,46  +/-2,63 | 1,08  +/-0,28 | 1,23  +/-0,60 | 4,54  +/-0,78 | 2,46  +/-1,94 |
| to run * | 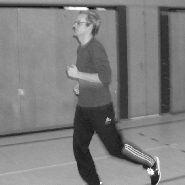 | rennen | 89,25 | 5,03  +/-1,42 | 3,39  +/-1,78 | 53,55 | 6,60  +/-0,65 | 3,00  +/-1,53 | 5,32  +/-1,33 | 6,93  +/-0,18 | 6,36  +/-0,68 | 4,96  +/-1,13 | 4,00  +/-1,78 |
| to smell * | 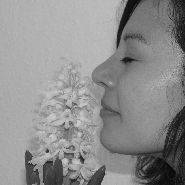 | riechen | 100,00 | 6,86  +/-0,36 | 1,57  +/-1,60 | 0,00 | 1,57  +/-0,65 | 5,00  +/-2,29 | 1,36  +/-0,63 | 1,00  +/-0,00 | 1,00  +/-0,00 | 5,07  +/-1,33 | 5,07  +/-1,44 |
| to roll | 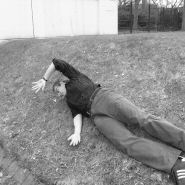 | rollen | 52,37 | 4,81  +/-1,77 | 4,12  +/-1,68 | 31,87 | 5,07  +/-1,23 | 2,43  +/-1,58 | 4,48  +/-1,55 | 4,57  +/-1,49 | 6,26  +/-0,94 | 1,71  +/-0,74 | 1,49  +/-0,76 |
| to stir | 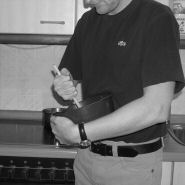 | ruehren | 85,70 | 6,71  +/-0,61 | 2,00  +/-1,84 | 0,00 | 2,93  +/-0,92 | 1,64  +/-1,39 | 5,57  +/-1,74 | 1,00  +/-0,00 | 1,50  +/-0,85 | 3,86  +/-0,77 | 3,21  +/-1,53 |
| to saw | 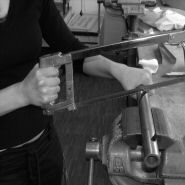 | saegen | 85,70 | 6,79  +/-0,43 | 2,00  +/-1,75 | 14,30 | 3,36  +/-0,84 | 1,50  +/-1,16 | 5,57  +/-1,83 | 1,79  +/-1,85 | 3,14  +/-1,70 | 2,86  +/-1,29 | 1,57  +/-1,02 |
| to vacuum * | 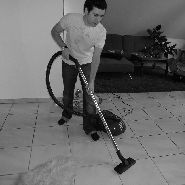 | saugen | 100,00 | 6,93  +/-0,27 | 1,64  +/-1,65 | 14,30 | 3,64  +/-0,84 | 1,50  +/-1,09 | 5,21  +/-1,72 | 4,00  +/-1,92 | 4,21  +/-1,19 | 4,79  +/-1,05 | 4,14  +/-0,86 |
| to play chess * | 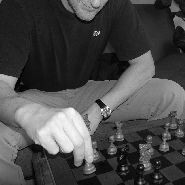 | schachspielen | 100,00 | 6,86  +/-0,36 | 1,21  +/-0,58 | 35,70 | 2,46  +/-1,27 | 3,07  +/-2,09 | 5,36  +/-1,55 | 1,00  +/-0,00 | 1,50  +/-0,76 | 3,50  +/-1,22 | 1,29  +/-0,83 |
| to make a gift * | 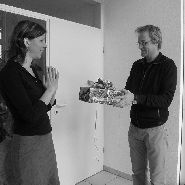 | schenken | 100,00 | 6,79  +/-0,43 | 1,79  +/-1,37 | 0,00 | 2,07  +/-0,83 | 3,07  +/-2,06 | 5,07  +/-2,02 | 1,14  +/-0,36 | 1,86  +/-0,77 | 3,43  +/-1,09 | 3,14  +/-1,35 |
| to shoot * | 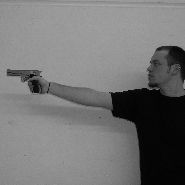 | schießen | 89,30 | 6,82  +/-0,40 | 1,54  +/-1,35 | 7,15 | 3,32  +/-1,29 | 2,86  +/-2,18 | 5,93  +/-1,17 | 2,25  +/-1,71 | 3,32  +/-1,66 | 1,71  +/-1,06 | 1,07  +/-0,27 |
| to sleep * | 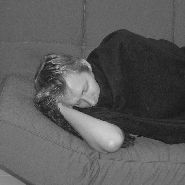 | schlafen | 100,00 | 7,00  +/-0,00 | 1,14  +/-0,53 | 7,10 | 1,86  +/-1,56 | 2,50  +/-2,38 | 2,29  +/-2,03 | 1,50  +/-0,94 | 2,57  +/-2,34 | 6,29  +/-0,99 | 5,86  +/-1,17 |
| to sneak * | 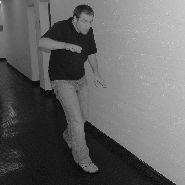 | schleichen | 60,70 | 5,82  +/-1,43 | 3,04  +/-1,55 | 42,85 | 4,54  +/-1,33 | 2,18  +/-1,31 | 3,14  +/-1,61 | 5,71  +/-1,32 | 5,46  +/-1,53 | 2,89  +/-0,95 | 2,00  +/-0,91 |
| to paint one’s face * | 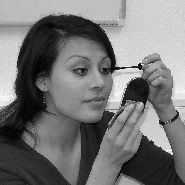 | schminken | 100,00 | 6,86  +/-0,36 | 1,64  +/-1,60 | 14,30 | 2,29  +/-0,73 | 5,43  +/-1,91 | 5,43  +/-1,83 | 1,00  +/-0,00 | 1,21  +/-0,43 | 4,79  +/-1,05 | 3,50  +/-2,03 |
| to cut * | 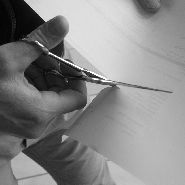 | schneiden | 100,00 | 6,86  +/-0,36 | 1,79  +/-1,63 | 14,30 | 2,21  +/-0,80 | 1,50  +/-1,09 | 5,14  +/-1,83 | 1,00  +/-0,00 | 1,14  +/-0,36 | 4,29  +/-1,07 | 4,00  +/-1,41 |
| to snorkel * | 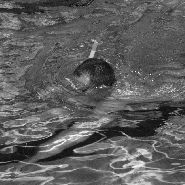 | schnorcheln | 71,40 | 5,71  +/-1,59 | 2,71  +/-1,77 | 53,80 | 5,64  +/-1,08 | 4,69  +/-2,18 | 5,64  +/-1,34 | 5,93  +/-1,33 | 6,29  +/-1,07 | 2,29  +/-0,83 | 1,29  +/-0,47 |
| to write * | 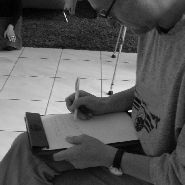 | schreiben | 100,00 | 6,93  +/-0,27 | 1,50  +/-1,61 | 0,00 | 2,36  +/-0,93 | 2,14  +/-1,70 | 5,29  +/-2,02 | 1,00  +/-0,00 | 1,21  +/-0,58 | 6,14  +/-0,95 | 6,21  +/-1,25 |
| to scream * | 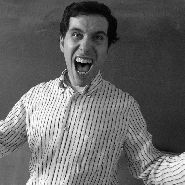 | schreien | 78,57 | 6,02  +/-1,58 | 2,52  +/-1,82 | 19,03 | 2,81  +/-1,12 | 6,40  +/-1,00 | 2,93  +/-1,42 | 1,14  +/-0,35 | 2,17  +/-1,33 | 3,86  +/-1,35 | 2,57  +/-1,54 |
| to hustle * | 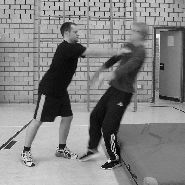 | schubsen | 85,70 | 6,64  +/-0,50 | 2,00  +/-1,62 | 21,40 | 4,21  +/-1,12 | 1,71  +/-1,07 | 5,93  +/-1,39 | 3,64  +/-1,74 | 4,79  +/-1,48 | 2,43  +/-0,94 | 1,43  +/-0,85 |
| to swim * | 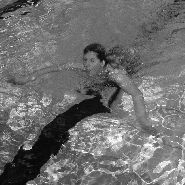 | schwimmen | 100,00 | 6,86  +/-0,35 | 1,64  +/-1,29 | 11,90 | 6,10  +/-0,64 | 3,86  +/-1,86 | 6,48  +/-0,67 | 6,38  +/-0,75 | 6,52  +/-0,61 | 3,43  +/-1,15 | 2,76  +/-1,68 |
| to swear * | 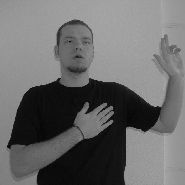 | schwoeren | 89,30 | 6,79  +/-0,43 | 1,46  +/-1,00 | 0,00 | 2,29  +/-1,11 | 3,79  +/-2,08 | 5,18  +/-1,66 | 1,07  +/-0,27 | 1,86  +/-1,22 | 1,96  +/-0,83 | 1,21  +/-0,42 |
| to sing | 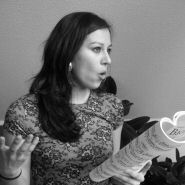 | singen | 50,00 | 5,93  +/-1,77 | 3,14  +/-1,99 | 28,60 | 2,50  +/-1,02 | 5,64  +/-1,65 | 2,57  +/-1,65 | 1,00  +/-0,00 | 1,86  +/-1,17 | 3,79  +/-0,97 | 3,36  +/-1,78 |
| to skate * | 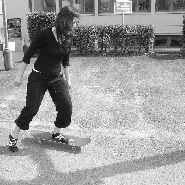 | skaten | 92,90 | 6,86  +/-0,35 | 1,57  +/-1,07 | 7,10 | 5,89  +/-0,96 | 2,36  +/-1,43 | 4,75  +/-1,31 | 6,43  +/-0,78 | 6,18  +/-0,98 | 3,32  +/-1,40 | 1,29  +/-0,61 |
| to speak * | 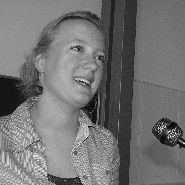 | sprechen | 42,88 | 4,61  +/-1,34 | 3,96  +/-1,50 | 50,00 | 2,45  +/-1,29 | 5,66  +/-1,69 | 3,23  +/-1,56 | 1,11  +/-0,27 | 1,45  +/-0,75 | 6,82  +/-0,40 | 6,71  +/-0,66 |
| to jump * | 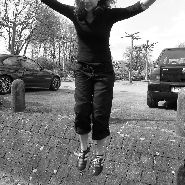 | springen | 100,00 | 6,82  +/-0,40 | 2,43  +/-1,06 | 21,40 | 5,86  +/-1,20 | 2,29  +/-1,40 | 4,07  +/-1,78 | 6,46  +/-0,94 | 6,29  +/-0,78 | 3,61  +/-1,31 | 2,71  +/-1,35 |
| to stamp * | 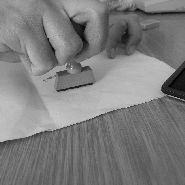 | stempeln | 100,00 | 6,93  +/-0,27 | 1,50  +/-1,61 | 7,10 | 2,00  +/-0,78 | 1,43  +/-0,94 | 4,50  +/-1,87 | 1,00  +/-0,00 | 1,21  +/-0,43 | 2,93  +/-1,00 | 1,29  +/-0,61 |
| to extend | 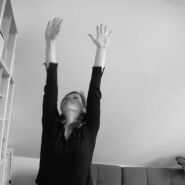 | strecken | 85,70 | 6,50  +/-0,76 | 2,29  +/-1,59 | 21,40 | 3,57  +/-1,09 | 1,93  +/-1,07 | 5,43  +/-1,16 | 2,71  +/-1,98 | 5,21  +/-1,58 | 4,43  +/-1,09 | 4,50  +/-1,61 |
| to paint * | 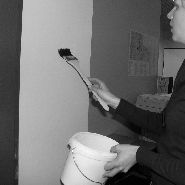 | streichen | 100,00 | 6,79  +/-0,43 | 1,86  +/-1,61 | 14,30 | 3,50  +/-0,94 | 1,57  +/-1,28 | 5,92  +/-1,38 | 1,79  +/-1,12 | 3,43  +/-1,70 | 2,71  +/-0,83 | 1,57  +/-0,76 |
| to knit * | 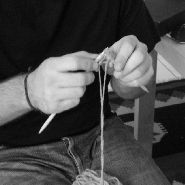 | stricken | 92,90 | 6,86  +/-0,36 | 1,50  +/-1,61 | 0,00 | 2,50  +/-0,94 | 2,29  +/-1,73 | 5,50  +/-1,79 | 1,00  +/-0,00 | 1,50  +/-0,65 | 2,50  +/-0,85 | 1,21  +/-0,58 |
| to search | 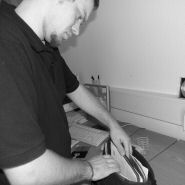 | suchen | 100,00 | 6,93  +/-0,27 | 1,36  +/-0,50 | 7,10 | 4,21  +/-1,58 | 3,93  +/-2,02 | 4,71  +/-1,33 | 4,21  +/-1,25 | 4,64  +/-1,60 | 4,64  +/-1,22 | 4,71  +/-1,94 |
| to dance * | 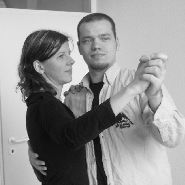 | tanzen | 85,70 | 6,21  +/-1,67 | 1,79  +/-1,25 | 35,70 | 6,29  +/-0,83 | 3,29  +/-1,64 | 6,21  +/-0,80 | 6,71  +/-0,47 | 6,71  +/-0,47 | 4,14  +/-1,10 | 3,86  +/-1,75 |
| to phone * | 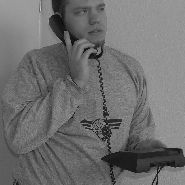 | telefonieren | 100,00 | 6,98  +/-0,09 | 1,33  +/-1,16 | 2,37 | 1,93  +/-0,97 | 5,10  +/-1,93 | 3,62  +/-1,77 | 1,02  +/-0,09 | 1,17  +/-0,43 | 6,10  +/-0,93 | 5,48  +/-1,10 |
| to type * | 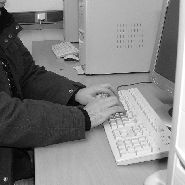 | tippen | 100,00 | 6,93  +/-0,27 | 1,07  +/-0,27 | 0,00 | 2,64  +/-1,34 | 1,79  +/-1,48 | 6,07  +/-1,38 | 1,00  +/-0,00 | 1,43  +/-0,85 | 5,71  +/-0,91 | 6,14  +/-1,17 |
| to carry * | 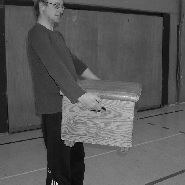 | tragen | 100,00 | 6,68  +/-0,55 | 1,75  +/-1,37 | 25,00 | 4,86  +/-1,30 | 2,18  +/-1,50 | 6,29  +/-1,14 | 4,11  +/-2,00 | 4,50  +/-1,69 | 5,39  +/-1,06 | 5,11  +/-1,20 |
| to kick * | 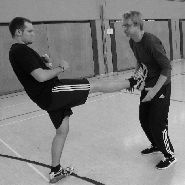 | treten | 92,90 | 6,43  +/-1,60 | 1,29  +/-0,47 | 23,10 | 5,93  +/-1,14 | 1,79  +/-1,25 | 3,29  +/-1,54 | 6,64  +/-0,75 | 6,07  +/-0,92 | 2,64  +/-0,84 | 1,50  +/-0,76 |
| to drum * | 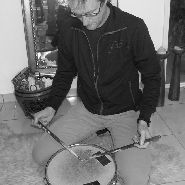 | trommeln | 97,63 | 6,93  +/-0,21 | 1,40  +/-1,27 | 7,13 | 4,24  +/-1,39 | 1,81  +/-1,40 | 6,12  +/-1,30 | 2,17  +/-1,91 | 2,86  +/-1,83 | 2,26  +/-1,08 | 1,29  +/-0,76 |
| to hug * | 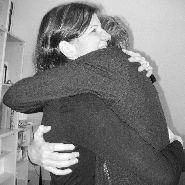 | umarmen | 100,00 | 7,00  +/-0,00 | 1,00  +/-0,00 | 0,00 | 4,00  +/-1,84 | 3,57  +/-1,60 | 6,21  +/-1,05 | 1,71  +/-0,91 | 5,00  +/-1,66 | 5,43  +/-0,94 | 4,79  +/-1,63 |
| to dress a wound * | 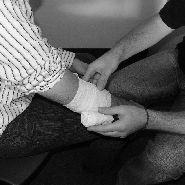 | verbinden | 100,00 | 7,00  +/-0,00 | 1,07  +/-0,27 | 0,00 | 3,14  +/-1,41 | 1,36  +/-0,84 | 5,71  +/-1,59 | 1,07  +/-0,27 | 1,79  +/-0,89 | 2,57  +/-1,34 | 1,14  +/-0,36 |
| to wash * | 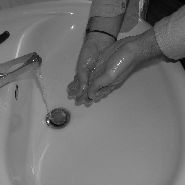 | waschen | 100,00 | 6,93  +/-0,27 | 1,14  +/-0,36 | 7,10 | 3,14  +/-1,56 | 1,71  +/-1,33 | 6,00  +/-1,47 | 1,36  +/-1,08 | 1,86  +/-1,29 | 6,64  +/-0,63 | 6,64  +/-0,63 |
| to cry * | 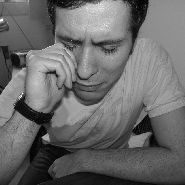 | weinen | 85,70 | 6,54  +/-0,97 | 1,86  +/-1,49 | 7,10 | 2,36  +/-0,89 | 5,50  +/-2,00 | 3,71  +/-1,79 | 1,00  +/-0,00 | 1,61  +/-1,00 | 3,68  +/-1,02 | 2,46  +/-1,44 |
| to throw | 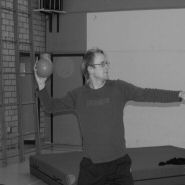 | werfen | 100,00 | 7,00  +/-0,00 | 1,21  +/-0,43 | 0,00 | 5,36  +/-1,01 | 2,43  +/-1,60 | 6,71  +/-0,61 | 3,57  +/-1,40 | 5,29  +/-0,99 | 4,43  +/-1,02 | 3,64  +/-1,69 |
| to wave to someone * | 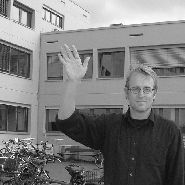 | winken | 89,30 | 6,68  +/-0,52 | 1,89  +/-1,20 | 28,55 | 3,00  +/-1,28 | 2,89  +/-1,98 | 5,61  +/-1,55 | 1,11  +/-0,32 | 1,46  +/-0,86 | 4,64  +/-1,57 | 4,25  +/-1,69 |
| to mop | 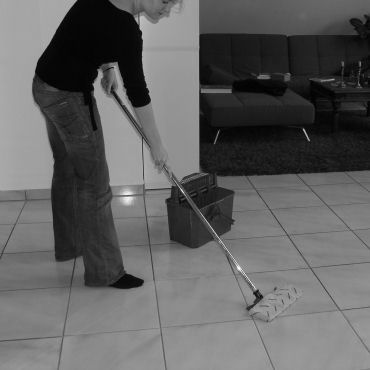 | wischen | 92,80 | 5,79  +/-2,01 | 2,50  +/-1,61 | 14,30 | 4,64  +/-1,15 | 1,93  +/-1,27 | 5,86  +/-1,35 | 4,21  +/-1,76 | 5,14  +/-1,17 | 4,93  +/-0,83 | 4,14  +/-1,17 |
| to choke * | 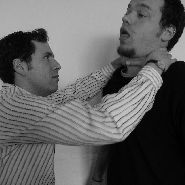 | wuergen | 92,90 | 6,36  +/-1,60 | 2,00  +/-1,80 | 14,30 | 3,43  +/-1,28 | 3,79  +/-2,19 | 6,29  +/-1,44 | 1,50  +/-0,65 | 2,50  +/-1,09 | 1,36  +/-0,63 | 1,00  +/-0,00 |
| to count * | 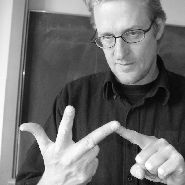 | zaehlen | 95,27 | 6,55  +/-0,89 | 1,76  +/-1,24 | 11,90 | 2,45  +/-1,18 | 3,60  +/-1,83 | 4,79  +/-1,76 | 1,00  +/-0,00 | 1,17  +/-0,39 | 4,86  +/-1,32 | 4,33  +/-1,64 |
