# Supplementary material for: Associative Vocabulary Learning: Development and Testing of Two Paradigms for the (Re-) Acquisition of Action- and Object-Related Words
Source: PLoS One. 2012 Jun 6;7(6):e37033. doi: 10.1371/journal.pone.0037033 (PMC3368912; doi:10.1371/journal.pone.0037033)
Supplement: Table S2 — Rating for Paradigm B – Rating objects. This is a list of all the objects rated for Paradigm B. On the questionnaire there were 4 items: 1 Please name the given item. 2 Please rate its recognizability from 1–7. (1 being best) 3 How strong is the depicted item associated with motion? (7 being most) 4 How strong are different body parts (arm/hand, leg/foot, head and whole body) associated with the object? (7 being most). Optionally raters could comment the pictures. (DOC) [file pone.0037033.s002.doc]

**Table S3 Rating for Paradigm B - Rating objects**

| **object**  **(* lexicon 1)** | **sample**  **picture** | **German term** | **1: naming conistency** | **2: Recog-nizability** | **3: association with motion** | **4: association to** | | | |
| --- | --- | --- | --- | --- | --- | --- | --- | --- | --- |
|  |  |  |  |  |  | **head** | **arm** | **leg** | **whole body** |
| barrel ***** | 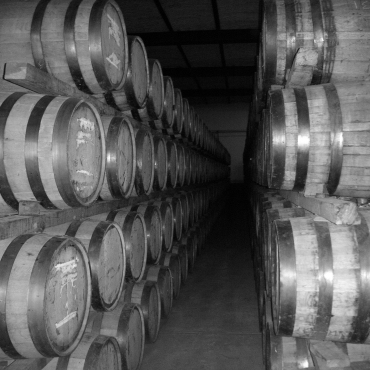 | Fass | 90,91% | 1,88  +/-1,11 | 1,67  +/-0,94 | 1,70  +/-1,16 | 1,75  +/-1,22 | 1,26  +/-0,89 | 1,69  +/-1,15 |
| book | 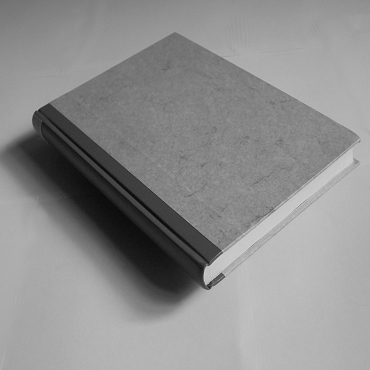 | Buch | 100% | 1,51  +/-0,75 | 1,67  +/-1,08 | 3,64  +/-1,86 | 2,74  +/-1,59 | 1,01  +/-0,05 | 1,19  +/-0,48 |
| bridge | 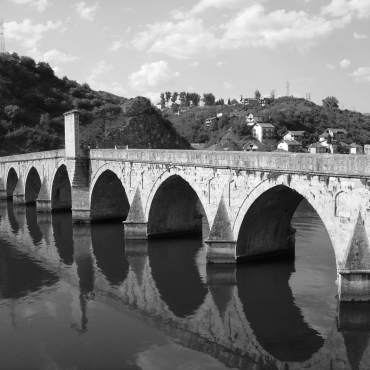 | Bruecke | 100% | 1,33  +/-0,64 | 3,22  +/-1,50 | 1,15  +/-0,44 | 1,36  +/-0,77 | 2,97  +/-1,91 | 2,84  +/-2,03 |
| buoy | 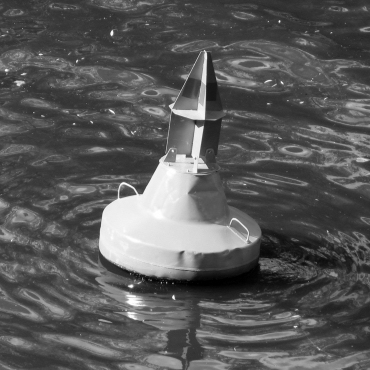 | Boje | 89,77% | 2,24  +/-1,34 | 3,32  +/-1,31 | 1,35  +/-0,79 | 1,49  +/-0,96 | 1,40  +/-0,81 | 1,84  +/-1,14 |
| cable | 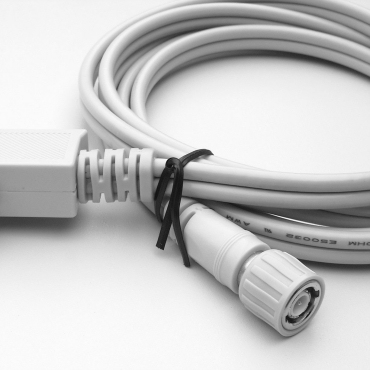 | Kabel | 98,86% | 1,89  +/-1,25 | 1,53  +/-0,72 | 1,24  +/-0,59 | 1,90  +/-1,05 | 1,10  +/-0,41 | 1,03  +/-0,13 |
| candle | 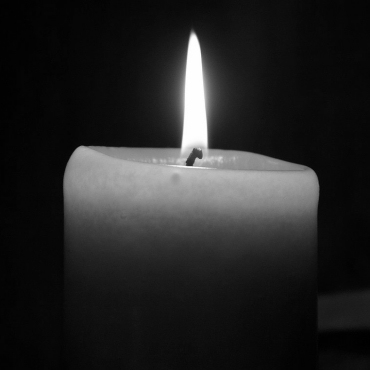 | Kerze | 96,59% | 1,65  +/-1,11 | 2,50  +/-1,04 | 1,90  +/-1,15 | 1,80  +/-1,09 | 1,23  +/-0,71 | 1,34  +/-0,76 |
| cardboard box ***** | 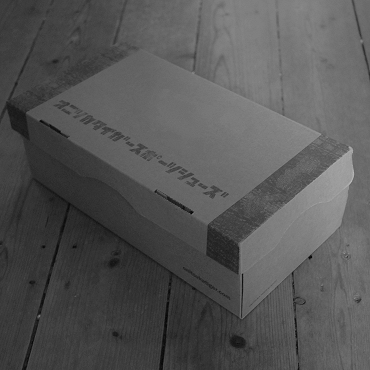 | Karton | 98,85% | 1,98  +/-1,24 | 1,67  +/-0,83 | 1,10  +/-0,30 | 2,22  +/-1,25 | 1,48  +/-1,03 | 1,44  +/-0,88 |
| castle | 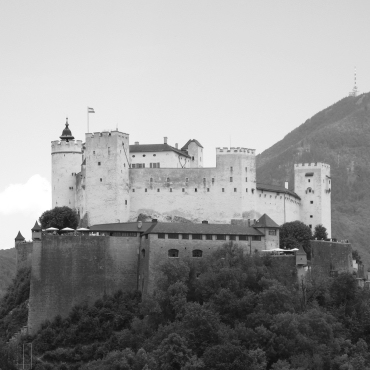 | Burg | 98,86% | 1,94  +/-1,17 | 1,33  +/-0,81 | 1,42  +/-0,86 | 1,39  +/-0,92 | 1,55  +/-1,05 | 2,01  +/-1,33 |
| chain | 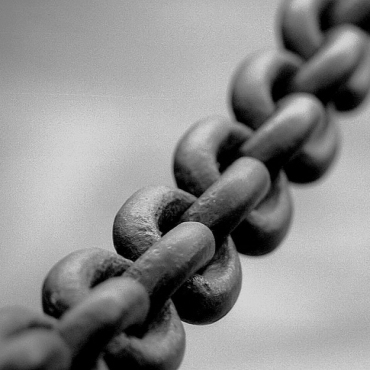 | Kette | 100% | 1,66  +/-1,10 | 1,71  +/-0,83 | 1,19  +/-0,64 | 1,99  +/-1,08 | 1,34  +/-0,68 | 1,25  +/-0,60 |
| church | 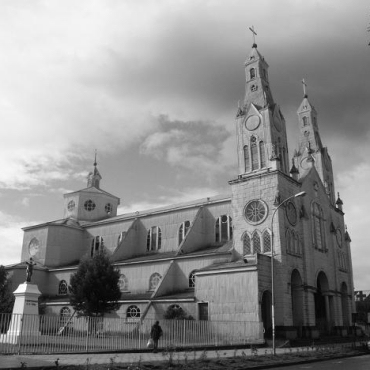 | Kirche | 96,55% | 1,43  +/-0,83 | 1,32  +/-0,72 | 1,60  +/-0,88 | 1,52  +/-1,02 | 1,33  +/-0,69 | 1,92  +/-1,51 |
| clock | 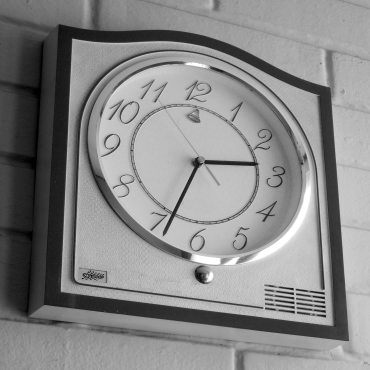 | Uhr | 93,18% | 1,88  +/-0,98 | 3,16  +/-1,87 | 2,31  +/-1,58 | 1,39  +/-0,84 | 1,06  +/-0,20 | 1,66  +/-1,43 |
| coffee  maker | 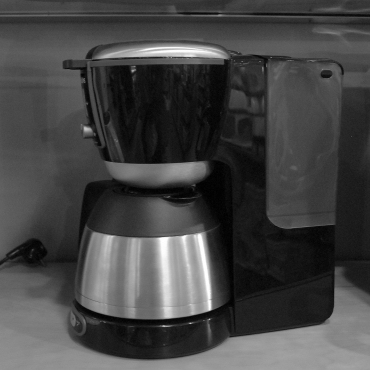 | Kaffee-maschine | 98,86% | 1,92  +/-1,05 | 1,91  +/-0,99 | 2,94  +/-1,86 | 2,30  +/-1,49 | 1,05  +/-0,18 | 1,48  +/-0,78 |
| crown | 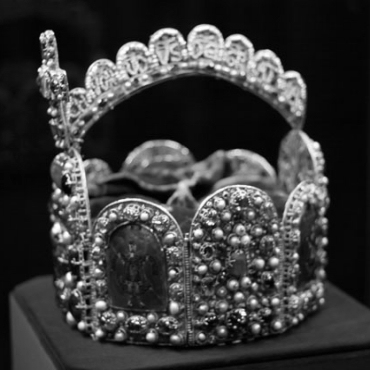 | Krone | 97,73% | 1,80  +/-1,01 | 1,56  +/-1,03 | 5,38  +/-1,85 | 1,69  +/-1,01 | 1,05  +/-0,18 | 1,76  +/-1,48 |
| cupboard * | 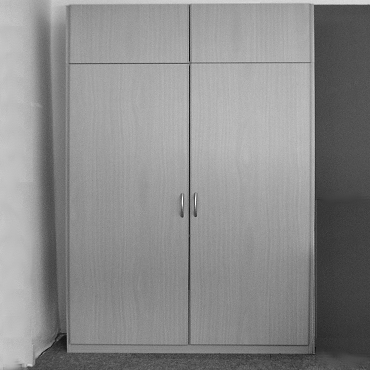 | Schrank | 89,77% | 1,80  +/-1,08 | 1,51  +/-1,10 | 1,31  +/-0,78 | 1,84  +/-1,11 | 1,18  +/-0,72 | 1,70  +/-1,16 |
| dustbin * | 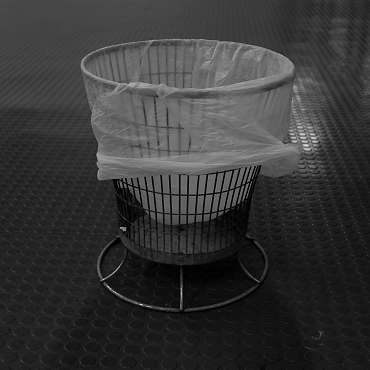 | Muelleimer | 98,86% | 1,53  +/-0,75 | 1,53  +/-0,76 | 1,11  +/-0,35 | 2,49  +/-1,31 | 1,23  +/-0,61 | 1,18  +/-0,47 |
| earth * | 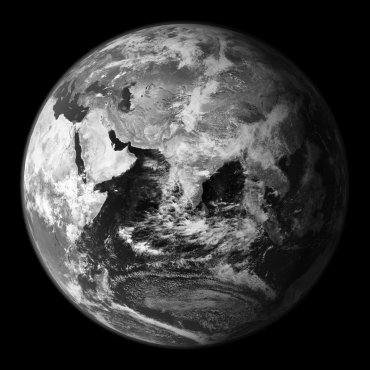 | Erde | 96,59% | 1,67  +/-1,05 | 4,24  +/-1,97 | 2,43  +/-1,90 | 1,57  +/-1,47 | 1,75  +/-1,61 | 2,96  +/-2,31 |
| field * | 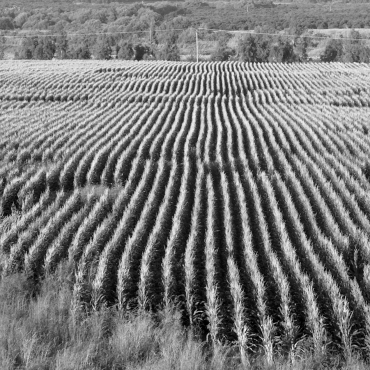 | Feld | 97,73% | 2,32  +/-1,39 | 2,41  +/-1,05 | 1,41  +/-1,14 | 2,14  +/-1,42 | 2,27  +/-1,58 | 2,00  +/-1,54 |
| flag | 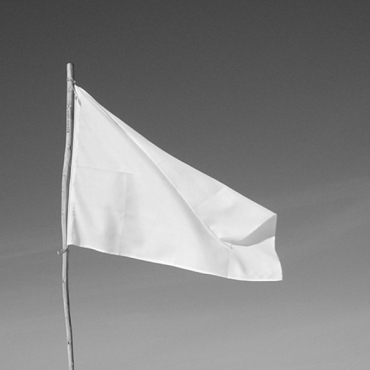 | Fahne | 96,59% | 1,67  +/-0,78 | 4,13  +/-1,48 | 1,33  +/-0,77 | 1,91  +/-1,05 | 1,05  +/-0,18 | 1,33  +/-0,68 |
| flush toilet | 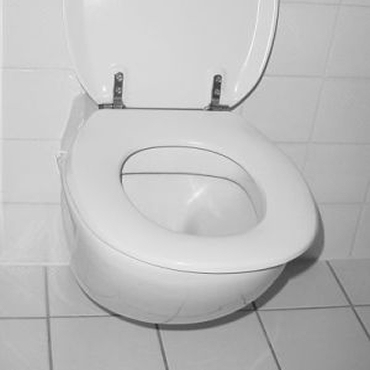 | Klo | 98,86% | 1,36  +/-0,62 | 2,36  +/-1,36 | 1,50  +/-1,05 | 1,70  +/-0,98 | 1,71  +/-1,18 | 3,52  +/-1,76 |
| gate | 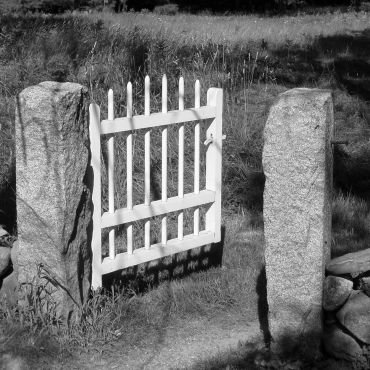 | Tor | 98,48% | 1,47  +/-0,80 | 2,41  +/-1,21 | 1,23  +/-0,68 | 2,20  +/-1,19 | 1,88  +/-1,26 | 1,91  +/-1,23 |
| gully | 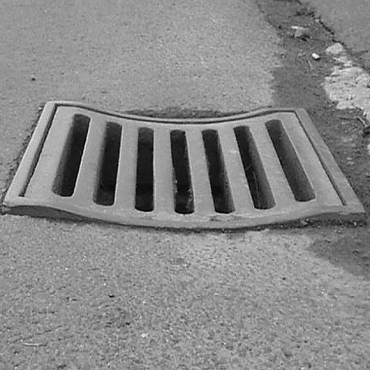 | Gully | 94,25% | 1,47  +/-0,70 | 1,61  +/-0,79 | 1,03  +/-0,13 | 1,31  +/-0,79 | 1,50  +/-0,81 | 1,23  +/-0,55 |
| hat * | 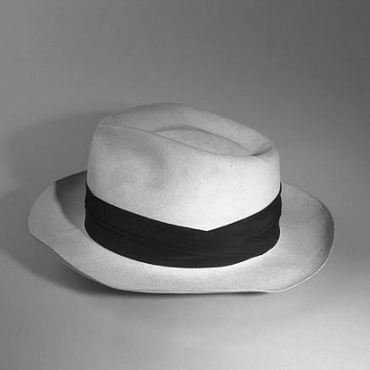 | Hut | 96,51% | 1,21  +/-0,61 | 1,60  +/-0,81 | 5,49  +/-1,81 | 1,55  +/-0,77 | 1,13  +/-0,44 | 1,38  +/-0,71 |
| heater | 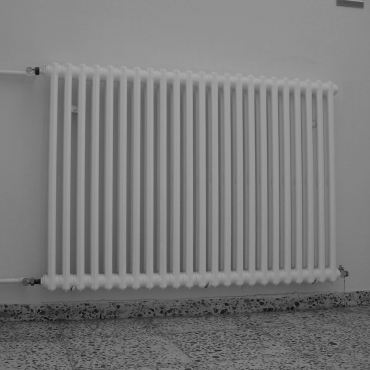 | Heizung | 100% | 1,68  +/-0,97 | 1,25  +/-0,43 | 1,19  +/-0,59 | 1,67  +/-1,08 | 1,34  +/-0,84 | 2,58  +/-1,65 |
| house * | 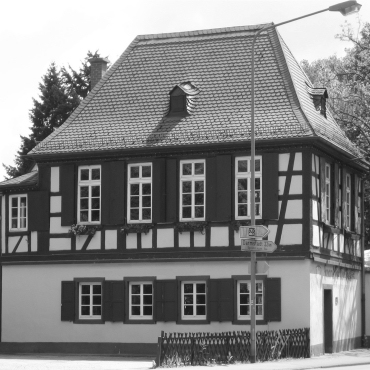 | Haus | 100% | 1,23  +/-0,52 | 1,23  +/-0,48 | 1,36  +/-0,79 | 1,41  +/-0,97 | 1,15  +/-0,41 | 2,18  +/-1,51 |
| hydrant * | 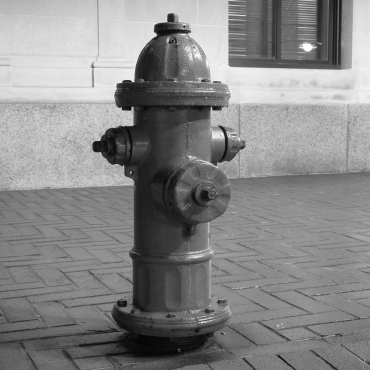 | Hydrant | 90,91% | 1,81  +/-1,18 | 1,89  +/-1,13 | 1,66  +/-1,43 | 1,90  +/-1,26 | 1,48  +/-1,04 | 1,82  +/-1,40 |
| island | 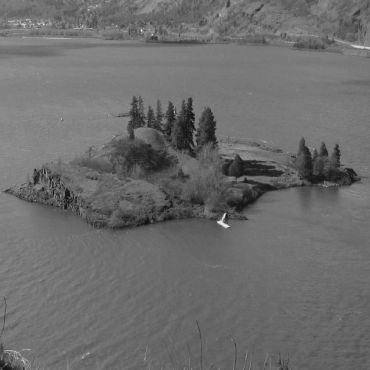 | Insel | 97,73% | 1,90  +/-0,96 | 1,82  +/-0,90 | 1,31  +/-0,73 | 1,42  +/-0,81 | 1,63  +/-0,95 | 1,91  +/-1,13 |
| lake | 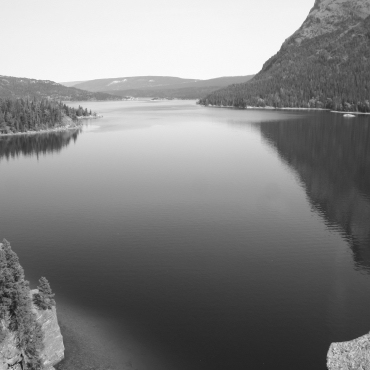 | See | 86,21% | 1,82  +/-1,16 | 3,10  +/-1,36 | 1,59  +/-1,17 | 2,23  +/-1,71 | 2,33  +/-1,71 | 3,17  +/-1,97 |
| lamp | 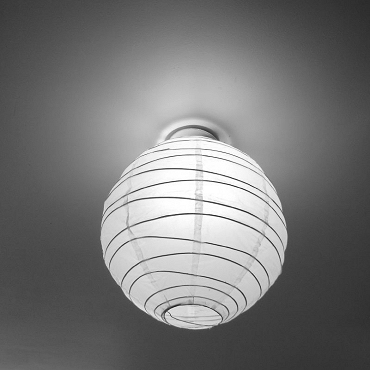 | Lampe | 98,86% | 1,53  +/-0,88 | 1,61  +/-1,14 | 1,92  +/-1,28 | 1,68  +/-0,99 | 1,08  +/-0,37 | 1,45  +/-0,99 |
| letter | 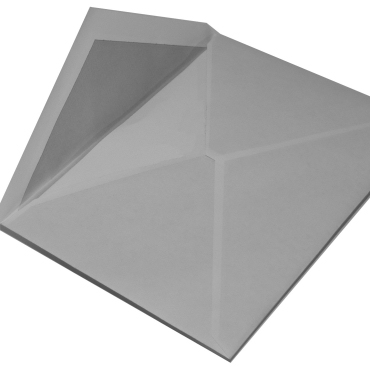 | Brief | 94,32% | 1,69  +/-1,01 | 2,61  +/-1,67 | 2,12  +/-1,46 | 2,95  +/-1,65 | 1,33  +/-0,88 | 1,15  +/-0,43 |
| loud-speaker | 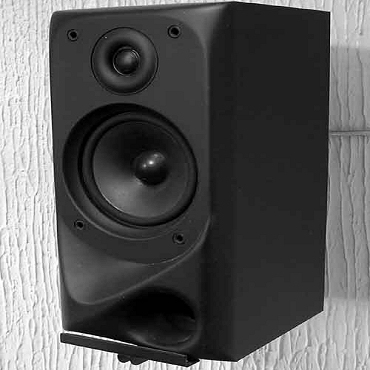 | Lautsprecher | 97,73% | 2,44  +/-1,49 | 1,74  +/-0,82 | 2,49  +/-1,54 | 1,27  +/-0,58 | 1,23  +/-0,61 | 1,53  +/-0,73 |
| moon * | 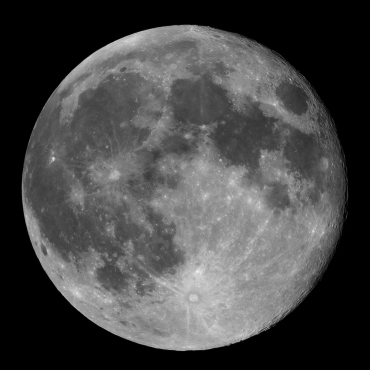 | Mond | 76,14% | 1,94  +/-1,27 | 3,45  +/-1,90 | 2,38  +/-1,80 | 1,15  +/-0,53 | 1,18  +/-0,61 | 2,25  +/-1,86 |
| paper | 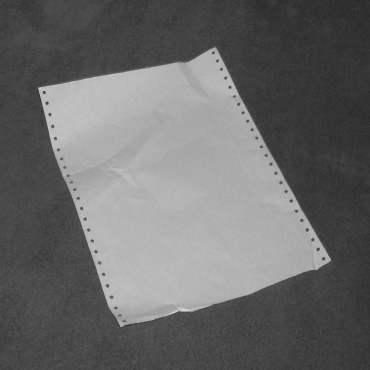 | Papier | 72,73% | 3,67  +/-1,69 | 1,92  +/-1,11 | 1,55  +/-0,96 | 2,33  +/-1,46 | 1,11  +/-0,47 | 1,14  +/-0,42 |
| personal computer | 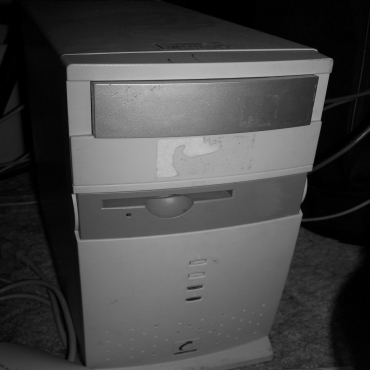 | Computer | 97,73% | 1,93  +/-1,11 | 1,58  +/-0,81 | 1,83  +/-1,15 | 1,82  +/-1,07 | 1,08  +/-0,34 | 1,18  +/-0,42 |
| pipe | 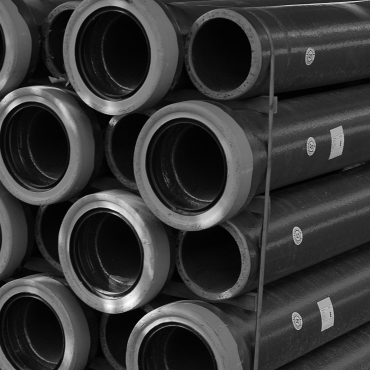 | Rohr | 97,73% | 2,53  +/-1,41 | 1,82  +/-1,09 | 1,11  +/-0,39 | 1,45  +/-0,80 | 1,46  +/-0,94 | 1,22  +/-0,51 |
| potato | 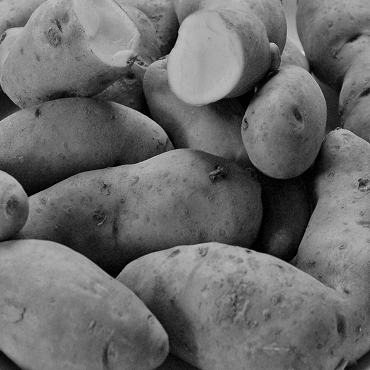 | Kartoffel | 98,86% | 1,89  +/-1,10 | 1,63  +/-0,79 | 3,40  +/-1,90 | 2,11  +/-1,18 | 1,10  +/-0,36 | 1,78  +/-1,06 |
| printer | 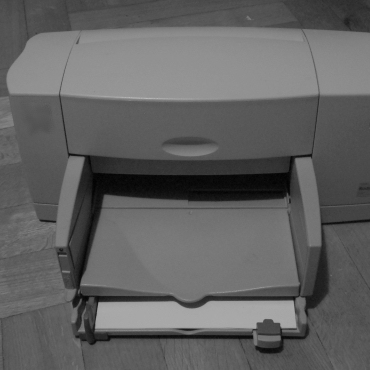 | Drucker | 94,19% | 1,60  +/-0,92 | 2,16  +/-1,13 | 1,36  +/-0,80 | 2,00  +/-1,30 | 1,01  +/-0,05 | 1,11  +/-0,32 |
| radio | 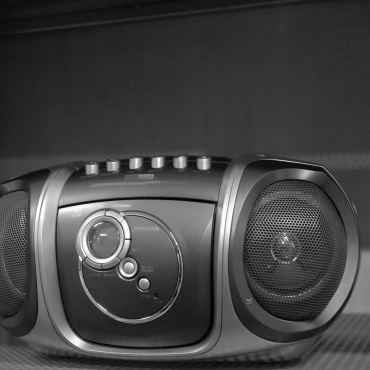 | Radio | 73,56% | 1,99  +/-1,17 | 1,90  +/-1,02 | 2,67  +/-1,57 | 1,77  +/-1,20 | 1,46  +/-1,09 | 1,59  +/-1,01 |
| rock | 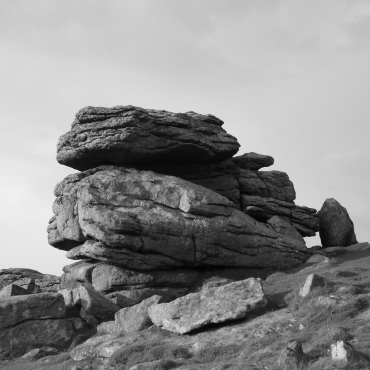 | Fels | 90,91% | 1,75  +/-0,99 | 1,47  +/-0,95 | 1,36  +/-0,77 | 1,59  +/-0,98 | 1,68  +/-1,11 | 2,07  +/-1,39 |
| shipping container | 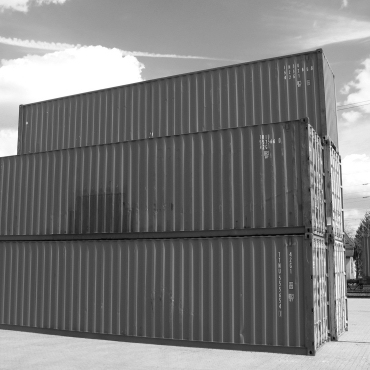 | Container | 97,70% | 1,66  +/-0,98 | 2,26  +/-1,36 | 1,08  +/-0,33 | 1,49  +/-1,02 | 1,20  +/-0,64 | 1,41  +/-0,77 |
| shrub * | 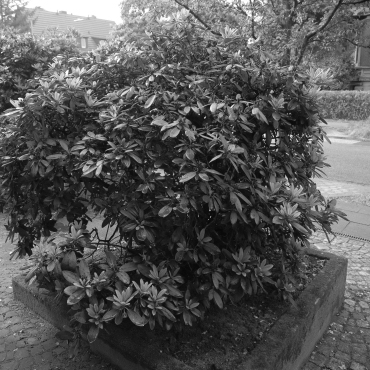 | Busch | 89,09% | 2,35  +/-1,32 | 2,11  +/-0,90 | 1,29  +/-0,55 | 1,54  +/-0,90 | 1,20  +/-0,58 | 1,43  +/-0,65 |
| stone | 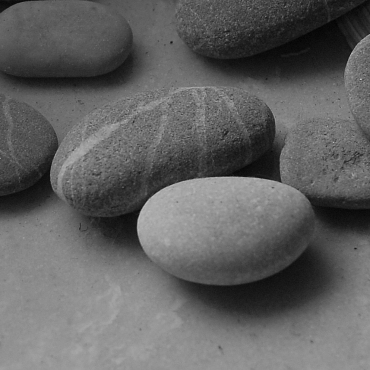 | Stein | 95,45% | 2,55  +/-1,61 | 1,55  +/-0,91 | 1,49  +/-0,92 | 1,89  +/-1,35 | 1,06  +/-0,23 | 1,33  +/-0,90 |
| sun * | 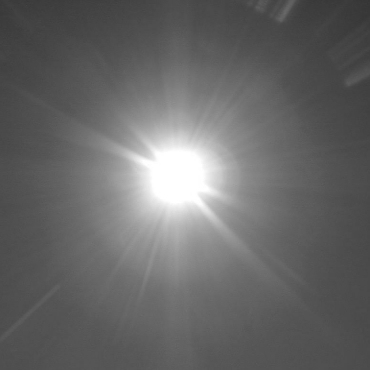 | Sonne | 91,82% | 2,31  +/-1,54 | 3,29  +/-1,76 | 2,84  +/-1,67 | 1,79  +/-1,19 | 1,64  +/-1,04 | 2,86  +/-1,89 |
| table * | 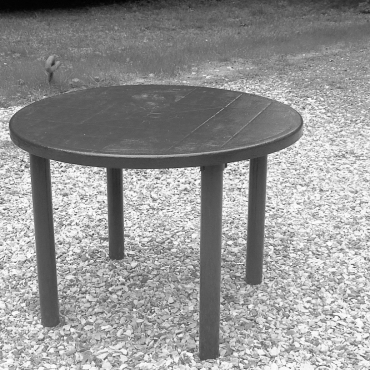 | Tisch | 81,82% | 1,63  +/-0,99 | 1,25  +/-0,62 | 1,29  +/-0,72 | 1,67  +/-1,04 | 1,82  +/-1,31 | 1,89  +/-1,17 |
| tart | 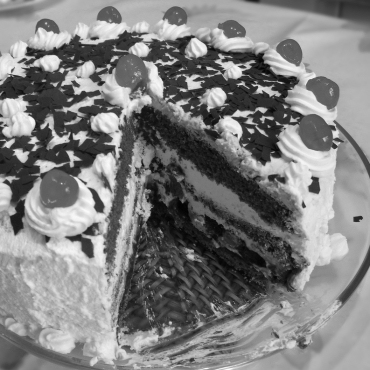 | Torte | 99,24% | 1,73  +/-1,05 | 1,59  +/-1,13 | 4,48  +/-2,05 | 2,33  +/-1,30 | 1,05  +/-0,21 | 1,71  +/-1,19 |
| television * | 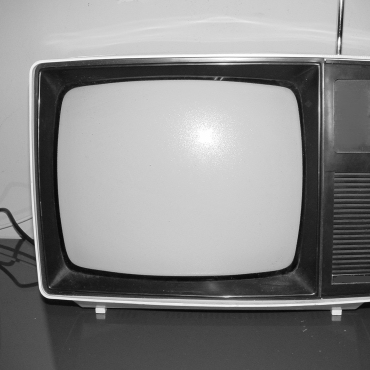 | Fernseher | 97,73% | 2,30  +/-1,26 | 2,07  +/-1,31 | 3,04  +/-1,69 | 1,34  +/-0,65 | 1,02  +/-0,11 | 1,20  +/-0,45 |
| tower * | 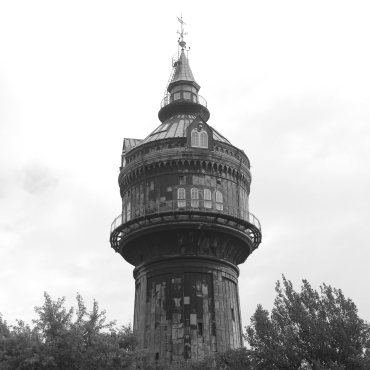 | Turm | 98,86% | 2,06  +/-1,19 | 1,39  +/-0,69 | 1,35  +/-0,80 | 1,15  +/-0,45 | 1,59  +/-1,09 | 1,83  +/-1,11 |
| tree | 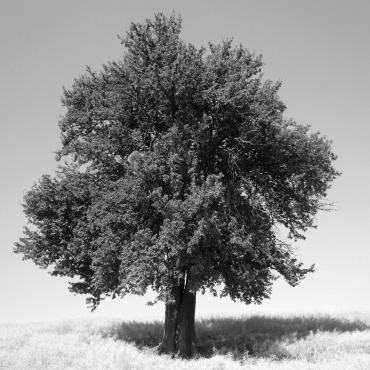 | Baum | 96,55% | 1,39  +/-0,81 | 2,33  +/-1,02 | 1,35  +/-0,79 | 1,80  +/-1,26 | 1,69  +/-1,24 | 1,86  +/-1,16 |
| vase | 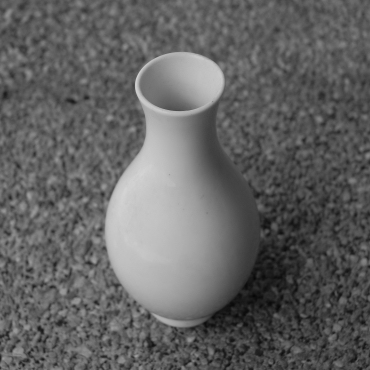 | Vase | 98,86% | 1,34  +/-0,69 | 1,27  +/-0,60 | 1,34  +/-0,74 | 1,84  +/-1,01 | 1,03  +/-0,13 | 1,60  +/-1,11 |
| wall | 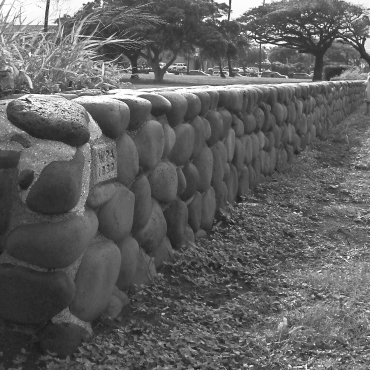 | Mauer | 97,70% | 1,96  +/-1,15 | 1,19  +/-0,47 | 1,17  +/-0,58 | 1,48  +/-0,98 | 1,23  +/-0,71 | 1,34  +/-0,76 |
| window * | 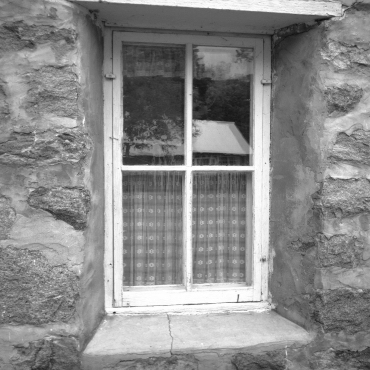 | Fenster | 98,86% | 1,49  +/-0,81 | 1,84  +/-1,11 | 1,66  +/-1,02 | 2,20  +/-1,38 | 1,05  +/-0,21 | 1,22  +/-0,56 |

Excluded items

| **object** | **example**  **picture** | **correct german term** | **1: naming conistency** | **2: Recognizability** | **3: association with motion** | **4: association to** | | | |
| --- | --- | --- | --- | --- | --- | --- | --- | --- | --- |
|  |  |  |  |  |  | **arm** | **leg** | **head** | **whole body** |
| antlers | 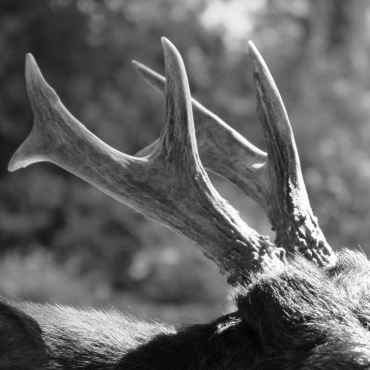 | Geweih | 92,05% | 2,01  +/-1,37 | 2,65  +/-1,25 | 3,58  +/-2,31 | 1,62  +/-1,11 | 1,46  +/-0,98 | 1,67  +/-1,15 |
| bacon | 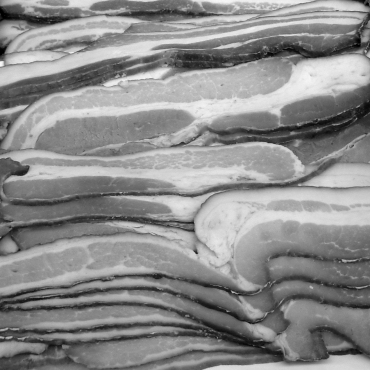 | Speck | 73,48% | 3,44  +/-1,87 | 1,66  +/-0,99 | 3,66  +/-2,13 | 1,81  +/-0,99 | 1,22  +/-0,66 | 1,98  +/-1,38 |
| beam/  joist | 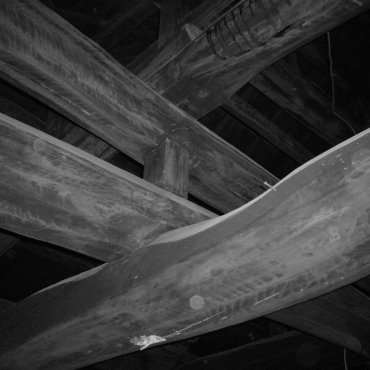 | Balken | 77,01% | 2,47  +/-1,62 | 1,28  +/-0,56 | 1,19  +/-0,54 | 1,60  +/-0,86 | 1,31  +/-0,79 | 1,14  +/-0,38 |
| carpet | 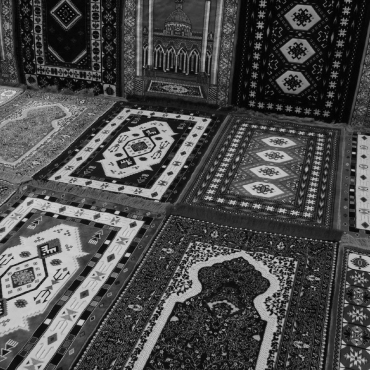 | Teppich | 93,02% | 1,81  +/-1,02 | 1,70  +/-1,11 | 1,27  +/-0,77 | 1,38  +/-1,01 | 2,19  +/-1,49 | 1,47  +/-0,94 |
| cloud | 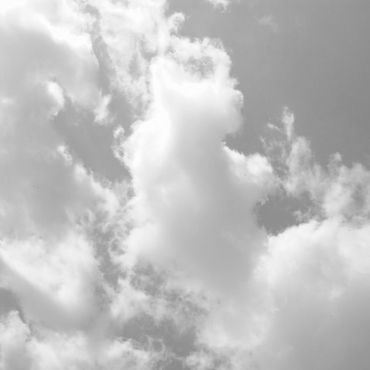 | Wolke | 77,27% | 2,39  +/-1,35 | 4,20  +/-1,52 | 2,06  +/-1,42 | 1,40  +/-0,81 | 1,34  +/-0,93 | 2,00  +/-1,37 |
| fence | 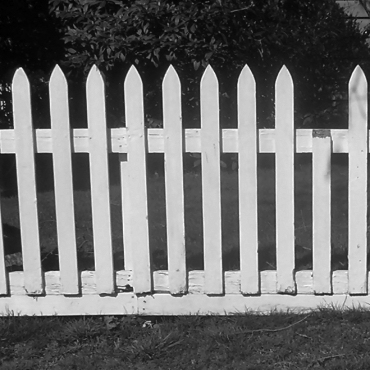 | Zaun | 93,10% | 1,63  +/-1,06 | 1,46  +/-0,99 | 1,14  +/-0,65 | 1,61  +/-1,23 | 1,49  +/-1,09 | 1,45  +/-1,16 |
| mountain | 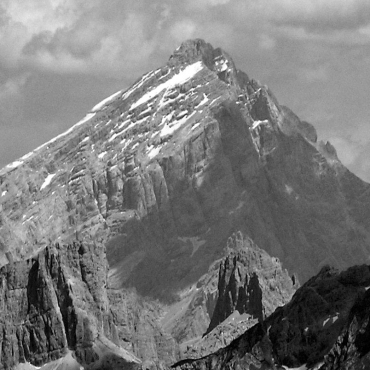 | Berg | 91,86% | 1,72  +/-1,16 | 1,89  +/-1,37 | 1,64  +/-1,08 | 1,84  +/-1,39 | 2,40  +/-1,67 | 2,54  +/-1,62 |
| sign | 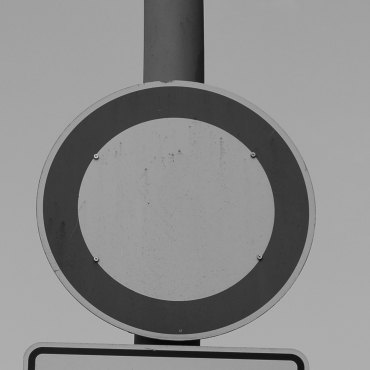 | Schild | 77,27% | 1,91  +/-1,32 | 2,23  +/-1,51 | 1,90  +/-1,32 | 1,44  +/-1,10 | 1,33  +/-0,80 | 1,44  +/-1,23 |
| star | 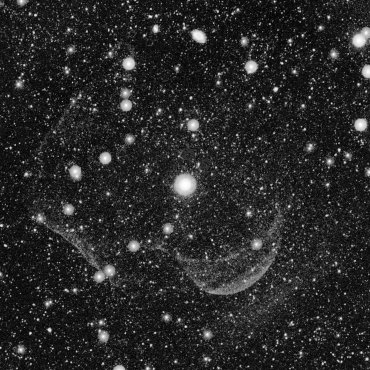 | Stern | 37,27% | 1,71  +/-1,13 | 3,63  +/-1,90 | 2,51  +/-1,80 | 1,10  +/-0,40 | 1,08  +/-0,38 | 1,86  +/-1,61 |
| tree branch | 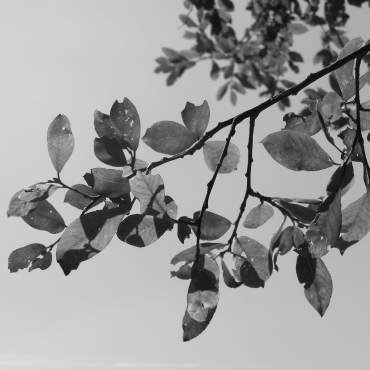 | Ast | 77,01% | 2,06  +/-1,02 | 2,67  +/-1,17 | 1,20  +/-0,61 | 1,98  +/-1,24 | 1,35  +/-0,78 | 1,20  +/-0,57 |
