# Supplementary material for: Associative Vocabulary Learning: Development and Testing of Two Paradigms for the (Re-) Acquisition of Action- and Object-Related Words
Source: PLoS One. 2012 Jun 6;7(6):e37033. doi: 10.1371/journal.pone.0037033 (PMC3368912; doi:10.1371/journal.pone.0037033)
Supplement: Table S3 — Rating for Paradigm B – Rating verbs. This is a list of all the verbs rated for Paradigm B. On the questionnaire there were 4 items:1 Please name the given item. 2 Please rate its recognizability from 1–7. (1 being best) 3 How strong is the depicted item associated with motion? (7 being most) 4 How strong are different body parts (arm/hand, leg/foot, head and whole body) associated with the verb? (7 being most). Optionally raters could comment the pictures. (DOC) [file pone.0037033.s003.doc]

**Table S3 Rating for Paradigm B - Rating verbs**

| action  (* lexicon 1) | sample  picture | German term | 1: naming consistency | 2: recogniz-ability | 3: association to motion | 4: association to | | | |
| --- | --- | --- | --- | --- | --- | --- | --- | --- | --- |
|  |  |  |  |  |  | head | arm | leg | whole body |
| to balance | 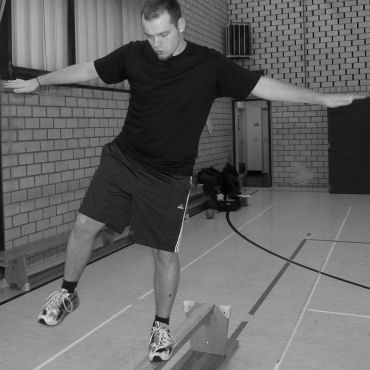 | balancieren | 96,74% | 1,80  +/-1,12 | 4,63  +/-1,52 | 3,22  +/-1,89 | 5,51  +/-1,38 | 5,74  +/-1,32 | 5,77  +/-1,52 |
| to box | 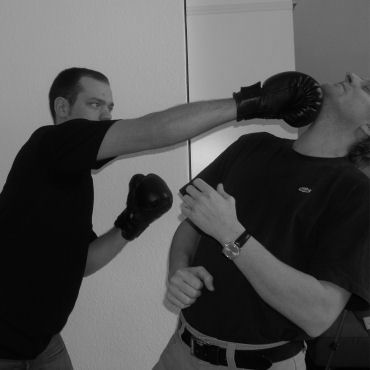 | boxen | 98,90% | 1,46  +/-0,84 | 5,60  +/-1,16 | 3,47  +/-1,92 | 6,39  +/-0,92 | 5,11  +/-1,72 | 5,39  +/-1,45 |
| to carry | 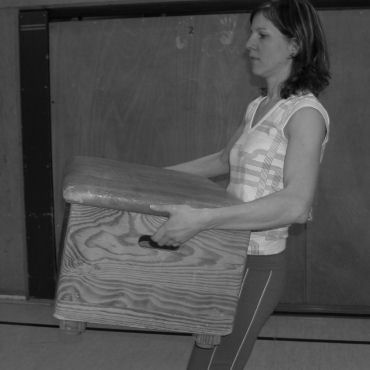 | tragen | 96,74% | 1,54  +/-0,85 | 4,13  +/-1,29 | 1,89  +/-1,41 | 6,01  +/-1,17 | 4,39  +/-1,76 | 4,35  +/-1,78 |
| to cheer * | 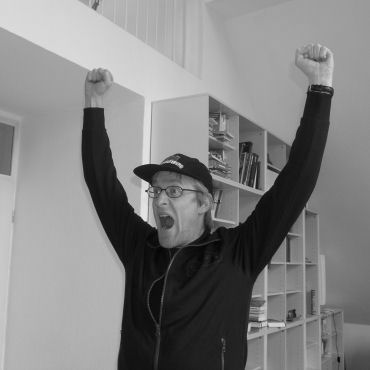 | jubeln | 90,11% | 2,25  +/-1,18 | 3,89  +/-1,45 | 4,21  +/-1,86 | 5,31  +/-1,27 | 3,38  +/-2,47 | 4,03  +/-1,69 |
| to clap | 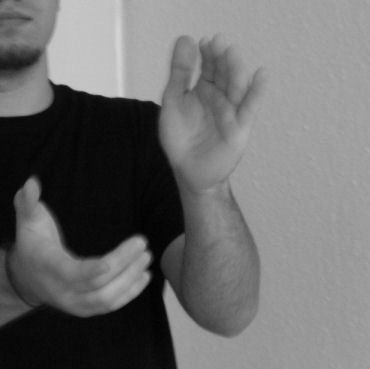 | klatschen | 96,74% | 2,15  +/-1,36 | 3,01  +/-1,23 | 1,75  +/-1,38 | 5,65  +/-1,45 | 1,14  +/-0,49 | 1,58  +/-0,93 |
| to climb * | 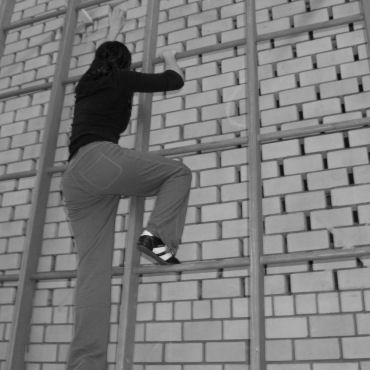 | klettern | 100% | 1,18  +/-0,47 | 5,41  +/-1,43 | 2,72  +/-1,67 | 6,11  +/-1,13 | 6,00  +/-1,19 | 5,79  +/-1,44 |
| to count * | 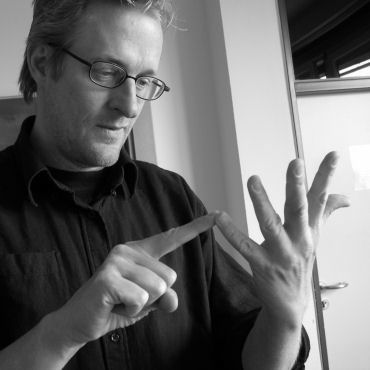 | zaehlen | 83,52% | 2,41  +/-1,34 | 2,06  +/-0,79 | 3,70  +/-1,98 | 4,27  +/-1,97 | 1,11  +/-0,40 | 1,20  +/-0,58 |
| to cut | 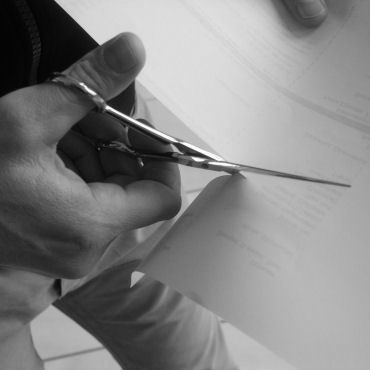 | schneiden | 100% | 1,70  +/-1,19 | 2,58  +/-0,97 | 1,91  +/-1,23 | 5,12  +/-1,73 | 1,10  +/-0,29 | 1,20  +/-0,42 |
| to dance | 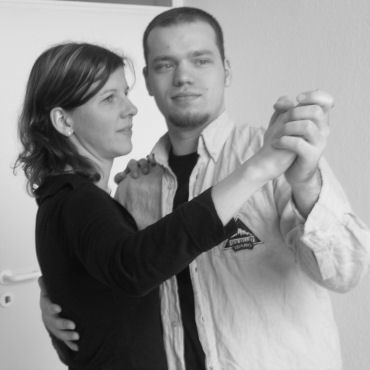 | tanzen | 100% | 1,55  +/-0,90 | 5,12  +/-1,47 | 2,91  +/-1,85 | 5,02  +/-1,36 | 5,97  +/-1,23 | 5,50  +/-1,43 |
| to do a puzzle | 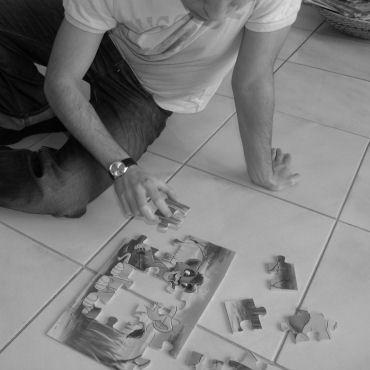 | puzzeln | 95,65% | 1,34  +/-0,70 | 1,97  +/-0,76 | 3,26  +/-2,02 | 4,49  +/-1,91 | 1,17  +/-0,44 | 1,34  +/-0,62 |
| to dress  (a wound) | 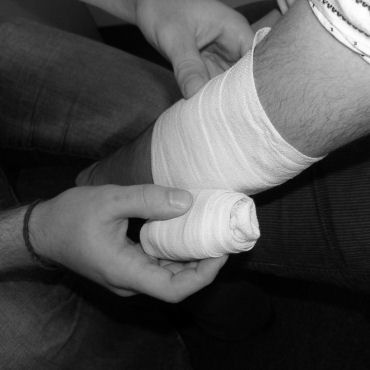 | verbinden | 95,65% | 1,87  +/-1,02 | 2,57  +/-0,98 | 2,17  +/-1,37 | 4,99  +/-1,61 | 1,43  +/-1,13 | 1,56  +/-1,13 |
| to drum | 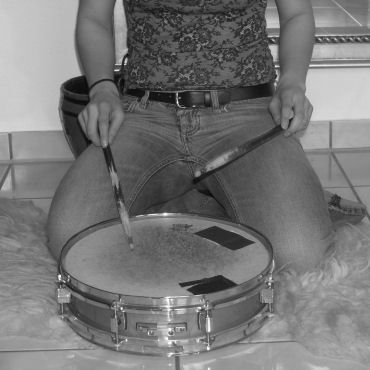 | trommeln | 98,91% | 1,28  +/-0,54 | 3,66  +/-1,41 | 2,35  +/-1,66 | 5,71  +/-1,34 | 1,88  +/-1,36 | 2,32  +/-1,45 |
| to eat | 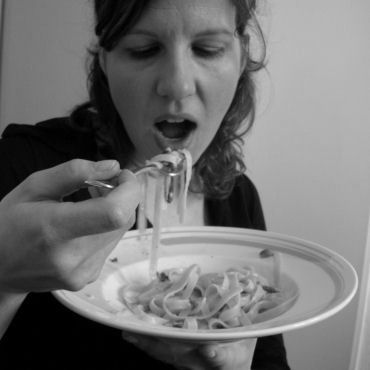 | essen | 98,90% | 1,27  +/-0,55 | 2,44  +/-0,93 | 5,35  +/-1,61 | 4,95  +/-1,71 | 1,13  +/-0,39 | 1,68  +/-1,11 |
| to fence * | 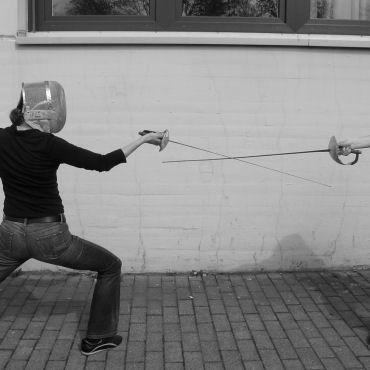 | fechten | 100% | 1,30  +/-0,67 | 5,41  +/-1,42 | 3,43  +/-1,91 | 6,18  +/-1,13 | 5,73  +/-1,32 | 5,67  +/-1,42 |
| to film | 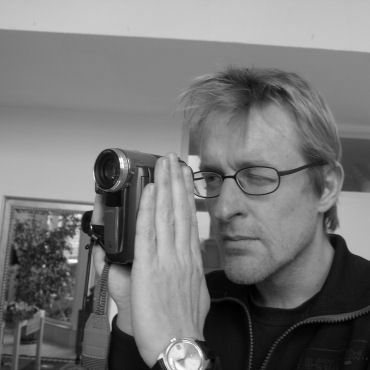 | filmen | 92,22% | 1,40  +/-0,72 | 2,07  +/-0,80 | 4,20  +/-1,81 | 4,57  +/-1,74 | 1,51  +/-0,79 | 1,61  +/-0,83 |
| to hang up * | 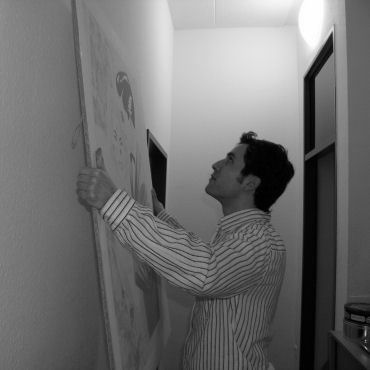 | aufhaengen | 91,30% | 2,40  +/-1,41 | 2,99  +/-1,18 | 2,44  +/-1,68 | 5,14  +/-1,52 | 2,32  +/-1,47 | 2,82  +/-1,70 |
| to hug | 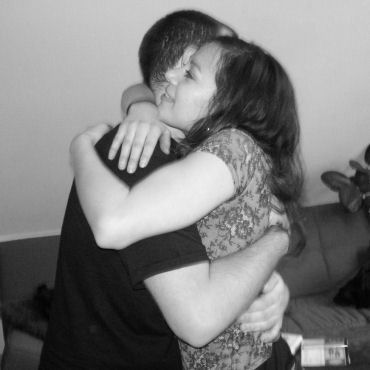 | umarmen | 96,70% | 1,46  +/-0,67 | 3,23  +/-1,58 | 2,66  +/-1,75 | 5,32  +/-1,54 | 2,17  +/-1,61 | 4,18  +/-1,91 |
| to iron | 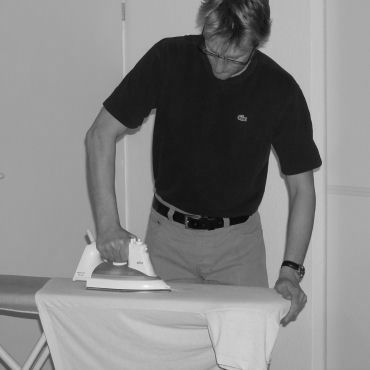 | buegeln | 100% | 1,26  +/-0,62 | 2,90  +/-1,26 | 2,07  +/-1,54 | 5,20  +/-1,65 | 1,99  +/-1,47 | 2,21  +/-1,42 |
| to juggle | 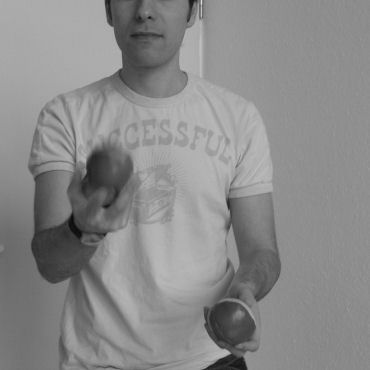 | jonglieren | 92,31% | 2,48  +/-1,55 | 4,37  +/-1,22 | 3,26  +/-1,73 | 6,09  +/-1,19 | 2,64  +/-1,50 | 3,50  +/-1,63 |
| to knit * | 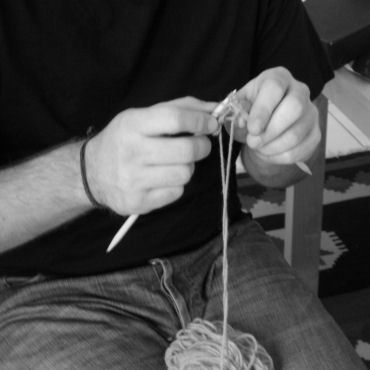 | stricken | 94,57% | 1,75  +/-1,05 | 2,65  +/-1,00 | 2,20  +/-1,57 | 5,59  +/-1,50 | 1,04  +/-0,14 | 1,28  +/-0,56 |
| to knock | 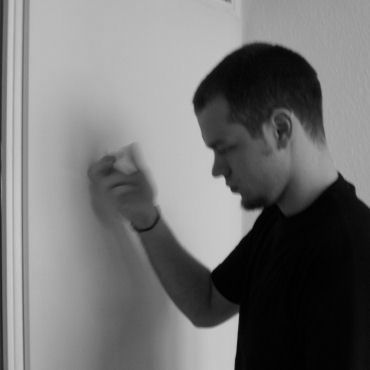 | klopfen | 100% | 1,78  +/-0,94 | 3,20  +/-1,01 | 1,90  +/-1,18 | 5,41  +/-1,54 | 2,44  +/-0,59 | 2,60  +/-0,81 |
| to massage | 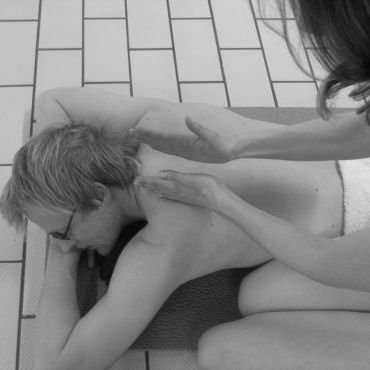 | massieren | 97,75% | 1,66  +/-1,03 | 3,56  +/-1,32 | 2,17  +/-1,61 | 5,75  +/-1,28 | 2,55  +/-1,99 | 3,47  +/-2,54 |
| to mop * | 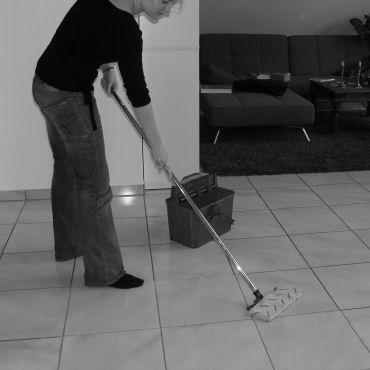 | wischen | 93,41% | 1,40  +/-0,72 | 3,75  +/-1,34 | 1,97  +/-1,58 | 5,46  +/-1,42 | 4,46  +/-1,64 | 4,25  +/-1,81 |
| to pack | 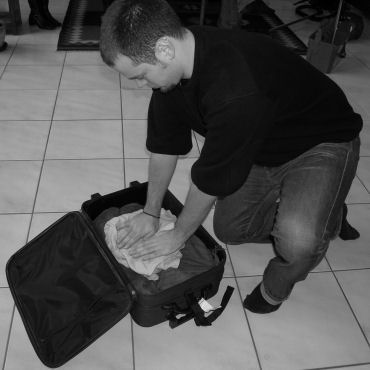 | packen | 96,74% | 1,95  +/-1,01 | 2,81  +/-0,91 | 2,24  +/-1,43 | 4,86  +/-1,64 | 2,16  +/-1,28 | 2,50  +/-1,39 |
| to paint | 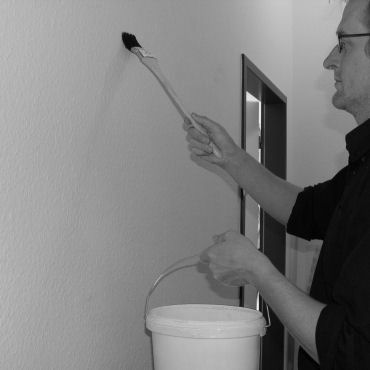 | streichen | 100% | 1,53  +/-0,91 | 3,45  +/-1,36 | 2,09  +/-1,46 | 5,42  +/-1,46 | 3,09  +/-1,75 | 3,40  +/-1,75 |
| to pinch | 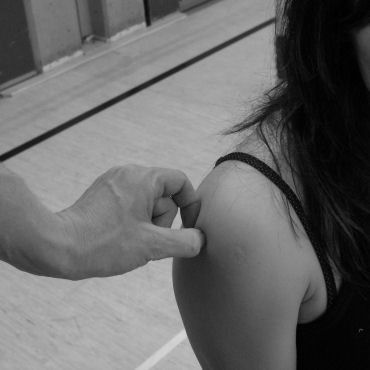 | kneifen | 94,57% | 2,13  +/-1,15 | 2,11  +/-0,79 | 1,63  +/-1,22 | 5,15  +/-1,74 | 1,15  +/-0,61 | 1,19  +/-0,51 |
| to play chess | 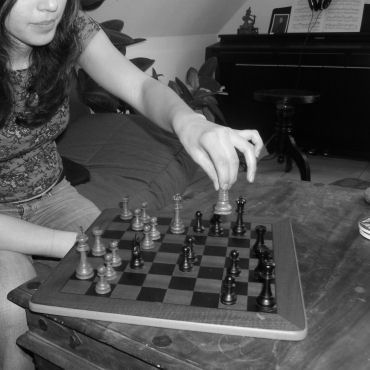 | Schach spielen | 98,90% | 1,35  +/-0,75 | 1,82  +/-0,67 | 4,87  +/-2,17 | 4,55  +/-1,97 | 1,07  +/-0,24 | 1,25  +/-0,65 |
| to pray | 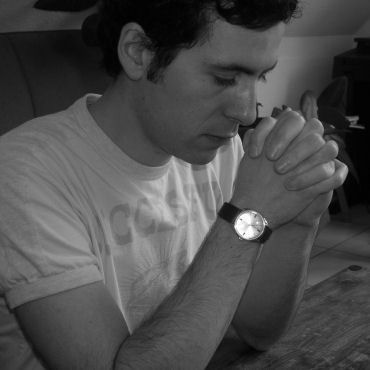 | beten | 89,13% | 1,93  +/-1,22 | 1,46  +/-0,56 | 3,84  +/-2,25 | 3,89  +/-2,08 | 1,09  +/-0,31 | 1,56  +/-1,10 |
| to pump | 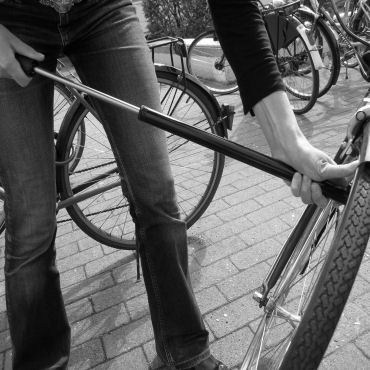 | pumpen | 100% | 1,36  +/-0,69 | 3,13  +/-1,19 | 1,67  +/-1,19 | 5,42  +/-1,44 | 1,95  +/-1,39 | 2,51  +/-1,61 |
| to punch /  perforate * | 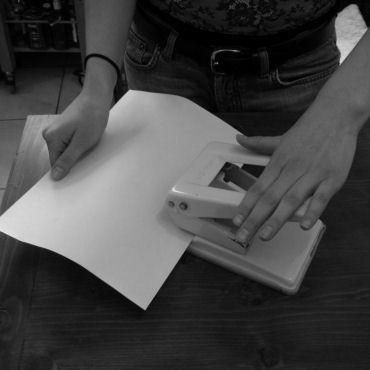 | lochen | 95,65% | 2,01  +/-1,22 | 2,15  +/-0,84 | 1,71  +/-1,34 | 4,76  +/-1,74 | 1,08  +/-0,26 | 1,55  +/-0,86 |
| to push | 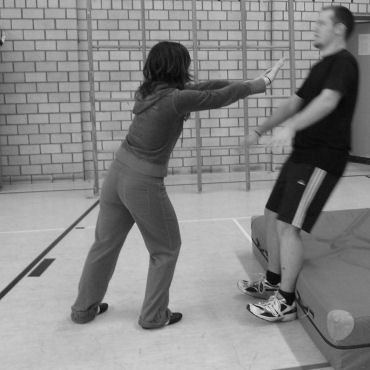 | schubsen | 85,87% | 1,95  +/-1,01 | 4,29  +/-1,22 | 2,17  +/-1,62 | 5,77  +/-1,15 | 4,07  +/-1,85 | 4,54  +/-1,81 |
| to ride a bike | 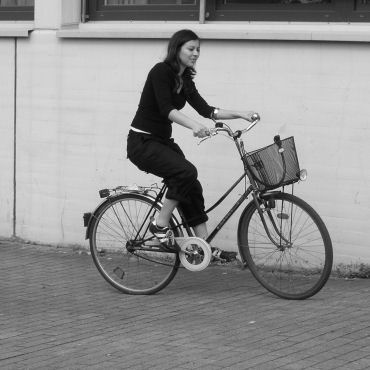 | fahren | 100% | 1,19  +/-0,48 | 5,35  +/-1,31 | 2,17  +/-1,44 | 4,67  +/-1,50 | 6,16  +/-1,16 | 5,09  +/-1,62 |
| to roll up | 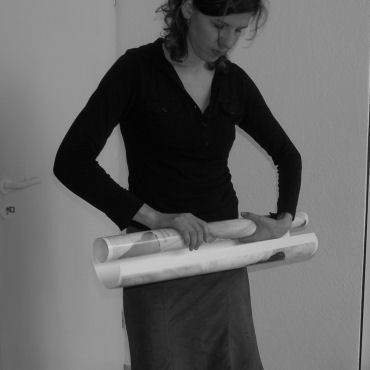 | einrollen | 97,83% | 1,54  +/-0,84 | 2,36  +/-0,78 | 1,74  +/-1,25 | 5,04  +/-1,45 | 1,27  +/-0,76 | 1,42  +/-0,68 |
| to run | 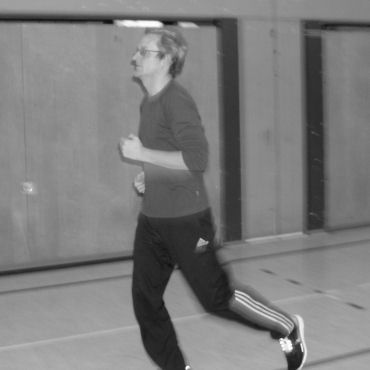 | rennen | 97,80% | 1,43  +/-0,61 | 5,90  +/-1,26 | 2,24  +/-1,54 | 4,80  +/-1,60 | 6,41  +/-1,02 | 5,64  +/-1,47 |
| to sew | 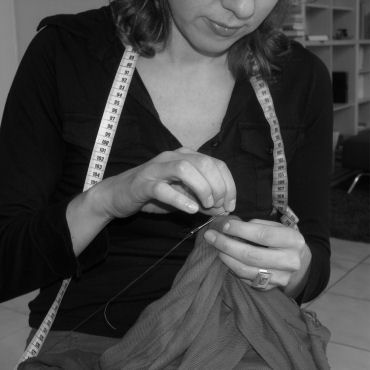 | naehen | 96,70% | 2,24  +/-1,52 | 2,31  +/-0,91 | 2,65  +/-1,75 | 5,25  +/-1,68 | 1,14  +/-0,36 | 1,25  +/-0,55 |
| to shave * | 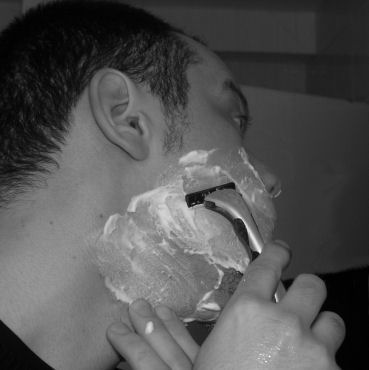 | rasieren | 100% | 1,67  +/-0,99 | 2,29  +/-0,81 | 4,76  +/-1,78 | 5,08  +/-1,69 | 1,18  +/-0,43 | 1,31  +/-0,65 |
| to shoot * | 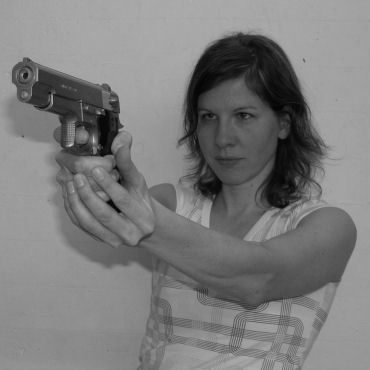 | schiessen | 92,39% | 1,38  +/-0,69 | 2,92  +/-1,22 | 3,28  +/-2,04 | 5,26  +/-1,60 | 2,25  +/-1,38 | 2,51  +/-1,60 |
| to signal (in class) | 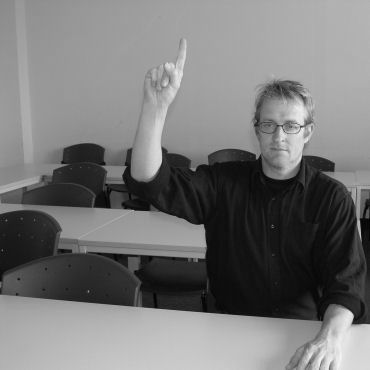 | melden | 94,57% | 1,49  +/-0,88 | 2,06  +/-0,75 | 1,99  +/-1,42 | 5,24  +/-1,70 | 1,15  +/-0,50 | 1,56  +/-1,04 |
| to smoke | 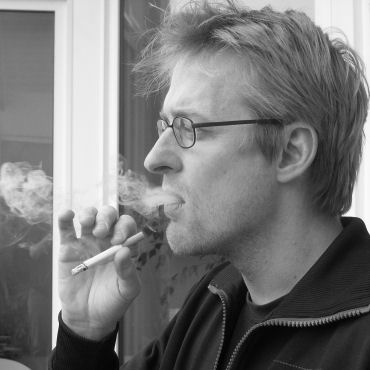 | rauchen | 100% | 1,14  +/-0,39 | 1,83  +/-0,73 | 4,55  +/-1,87 | 4,18  +/-1,90 | 1,17  +/-0,65 | 1,50  +/-0,86 |
| to stamp * |  | stempeln | 94,38% | 2,59  +/-1,55 | 2,09  +/-0,77 | 1,90  +/-1,43 | 4,98  +/-1,70 | 1,10  +/-0,39 | 1,18  +/-0,49 |
| to stretch | 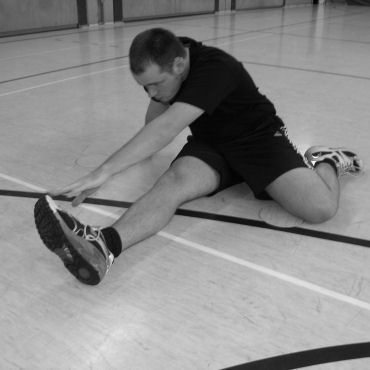 | dehnen | 100% | 1,52  +/-0,73 | 3,88  +/-1,64 | 1,90  +/-1,47 | 5,02  +/-1,58 | 5,16  +/-1,58 | 5,45  +/-1,54 |
| to swim * | 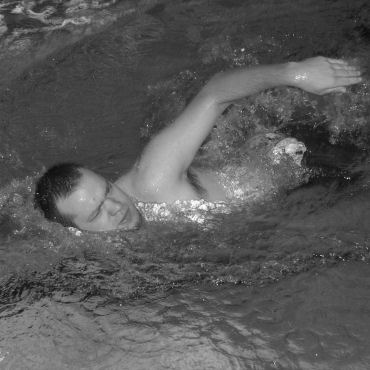 | schwimmen | 98,91% | 1,83  +/-1,22 | 5,96  +/-1,27 | 3,54  +/-1,97 | 6,17  +/-1,05 | 6,17  +/-1,09 | 5,92  +/-1,38 |
| to take a photo | 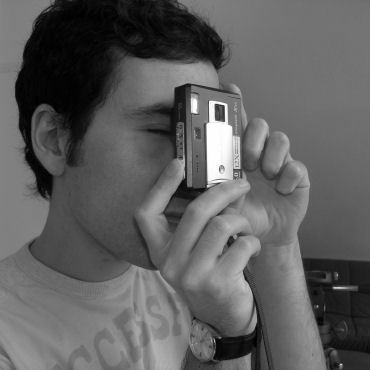 | fotografieren | 98,91% | 1,32  +/-0,69 | 2,02  +/-0,86 | 3,99  +/-1,92 | 4,71  +/-1,76 | 1,26  +/-0,67 | 1,35  +/-0,74 |
| to tie sth | 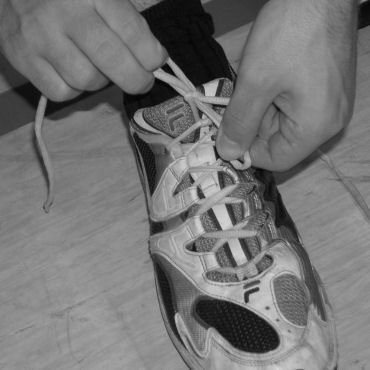 | binden | 90,00% | 2,03  +/-1,01 | 2,14  +/-0,94 | 2,36  +/-2,04 | 4,84  +/-1,92 | 2,02  +/-1,52 | 1,91  +/-1,49 |
| to type * | 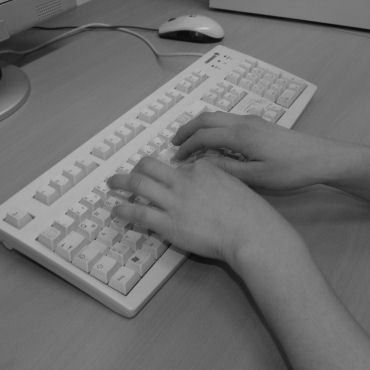 | tippen | 100% | 1,33  +/-0,62 | 2,23  +/-0,89 | 2,69  +/-1,90 | 5,26  +/-1,77 | 1,07  +/-0,25 | 1,23  +/-0,61 |
| to vacuum | 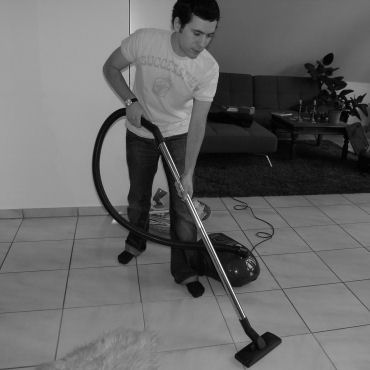 | saugen | 98,91% | 1,27  +/-0,64 | 3,75  +/-1,32 | 1,85  +/-1,47 | 5,21  +/-1,59 | 4,51  +/-1,57 | 4,00  +/-1,72 |
| to wash | 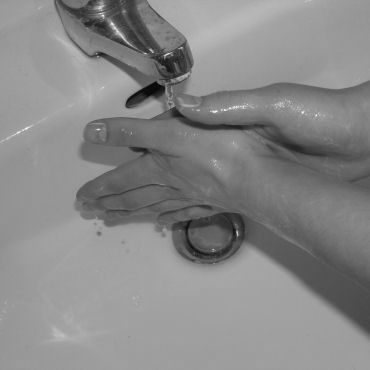 | waschen | 100% | 1,53  +/-1,03 | 2,38  +/-1,00 | 1,51  +/-0,84 | 5,28  +/-1,71 | 1,20  +/-0,53 | 1,32  +/-0,67 |
| to wave to someone * | 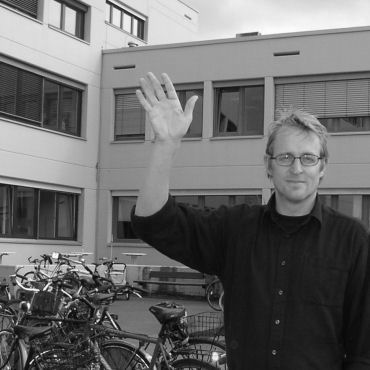 | winken | 94,38% | 1,59  +/-0,90 | 2,60  +/-0,99 | 2,12  +/-1,31 | 5,04  +/-1,75 | 1,28  +/-0,64 | 1,66  +/-0,99 |
| to whistle * | 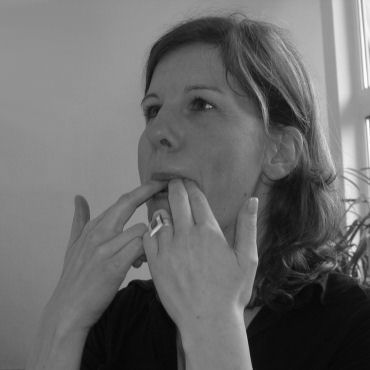 | pfeifen | 97,83% | 1,51  +/-0,81 | 1,93  +/-0,86 | 5,04  +/-1,79 | 4,64  +/-1,76 | 1,07  +/-0,23 | 1,13  +/-0,43 |
| to write * | 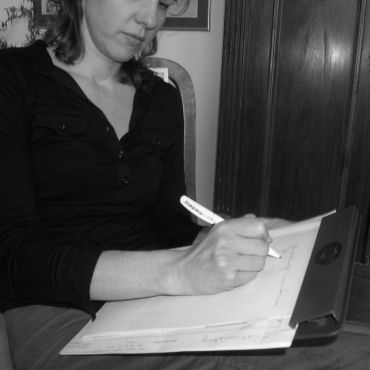 | schreiben | 100% | 1,77  +/-1,00 | 2,08  +/-0,69 | 2,97  +/-1,80 | 5,09  +/-1,73 | 1,09  +/-0,32 | 1,16  +/-0,36 |
| from paradigm B excluded items | | | | | | | | | |
| **verb** | **example**  **picture** | **correct german term** | **1: naming consistency** | **2: recogniz-ability** | **3: association to motion** | **4: association to** | | | |
|  |  |  |  |  |  | **head** | **arm** | **Head** | **Whole body** |
| to be cold | 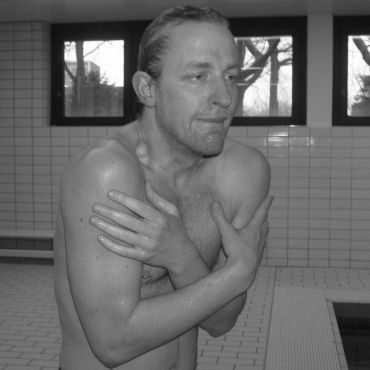 | frieren | 96,74% | 2,11  +/-1,40 | 2,39  +/-1,11 | 2,29  +/-1,44 | 3,27  +/-1,93 | 2,71  +/-1,77 | 4,49  +/-1,99 |
| to choke so | 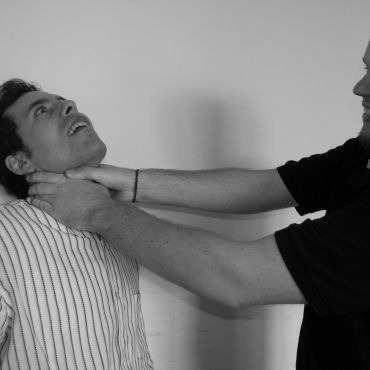 | wuergen | 98,91% | 1,52  +/-0,93 | 3,68  +/-1,27 | 3,16  +/-2,05 | 5,83  +/-1,24 | 2,00  +/-1,37 | 2,91  +/-1,79 |
| to fall | 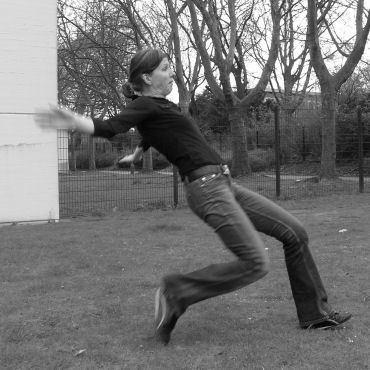 | fallen | 71,74% | 3,24  +/-1,87 | 4,32  +/-1,38 | 2,31  +/-1,48 | 4,51  +/-1,68 | 4,90  +/-1,66 | 5,20  +/-1,59 |
| to skate | 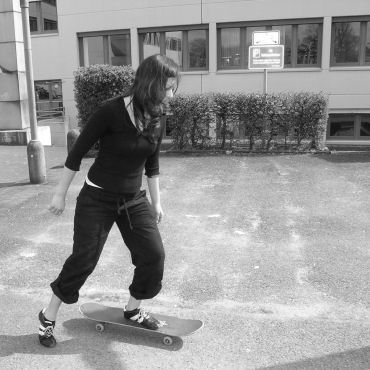 | skaten | 94,57% | 1,16  +/-0,38 | 5,33  +/-1,37 | 2,60  +/-1,75 | 4,76  +/-1,63 | 5,99  +/-1,17 | 5,65  +/-1,38 |
| to snorkel | 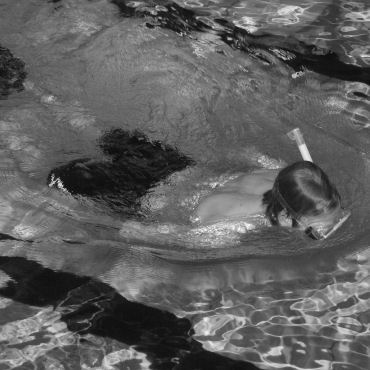 | schnorcheln | 81,32% | 2,42  +/-1,68 | 5,24  +/-1,47 | 3,92  +/-2,04 | 5,74  +/-1,37 | 5,78  +/-1,43 | 5,66  +/-1,51 |
| to swear | 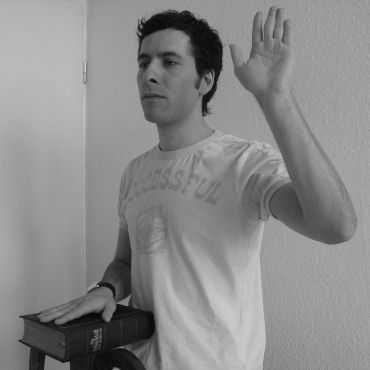 | schwoeren | 73,91% | 2,16  +/-1,50 | 1,84  +/-0,70 | 3,18  +/-1,77 | 4,45  +/-1,92 | 1,24  +/-0,82 | 1,37  +/-0,89 |
| to threaten | 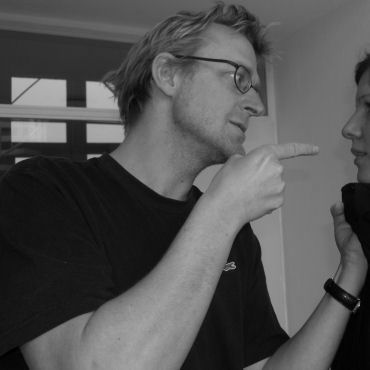 | drohen | 57,61% | 2,63  +/-1,41 | 2,48  +/-0,98 | 4,00  +/-1,67 | 4,36  +/-1,55 | 1,65  +/-1,08 | 2,55  +/-1,71 |
