# Supplementary material for: Associative Vocabulary Learning: Development and Testing of Two Paradigms for the (Re-) Acquisition of Action- and Object-Related Words
Source: PLoS One. 2012 Jun 6;7(6):e37033. doi: 10.1371/journal.pone.0037033 (PMC3368912; doi:10.1371/journal.pone.0037033)
Supplement: Table S4 — Results of neuropsychological testing. (DOC) [file pone.0037033.s004.doc]

# Table S4 - Results of neuropsychological testing:

|  | Paradigm A | Paradigm B |
| --- | --- | --- |
| Age | 24.95± 0.70 (21-34) | 26.20± 0.74 (24-32) |
| No. of foreign languages | 1.90± 0.26 (1-3) | 2.20± 0.29 (1-4) |
| D2-Test (182.8± 38,5) | 185.00± 8.17 (101-240) | 206.10± 5.82 (170-237) |
| BDI (6,45± 5,2) | 2.97± 0.75 (0-9) | 2.80±1.17 (0-10) |
| VLMT DG 1-5 (55.94± 7.57) | 66.95± 1.08 (54-72) | 64.60± 1.02 (58-69) |
| VLMT DG 7 (12.13± 2.24) | 14.37± 0.27 (11-15) | 14.40± 0.27 (13-15) |
| VLMT DG5-DG7 (1.29± 1.84) | 0.42± 0.16 (0-2) | 0.50± 0.27 (0-2) |
| VLMT W-F (13.86± 1.46) | 14.63± 0.18 (13-15) | 14.90± 0.32 (14-15) |
| digit spans | 16.63± 0.72 (11-21) | 16.10± 0.91 (10-20) |
| logical reasoning (22.9± 5.2) | 26.21± 1.25 (16-43) | 26.30± 1.27 (18-32) |
| ROCFT copy (29,4± 7) | 35.95± 0.53 (35-36) | 35.70± 0.30 (33-36) |
| ROCFT memory (35 ±17.3) | 49.39± 2.78 (19-63) | 52.9± 1.99(44-64) |
| ROCFT  memory quotient (120± 52) | 137.39± 7.7 (52.78-175) | 148.12± 5.24 (122.22-177.78) |
| verbal fluency: semantic subtest  (median: 43) | 38.58±1.68 (24-50) | 43.60±2.63 (31-57) |
| verbal fluency: formal subtest  (median: 29) | 25.73± 1.7 (14-38) | 30.70± 1.9 (20-39) |
